# Fifth European *Dirofilaria* and *Angiostrongylus* Days (FiEDAD) 2016: Vienna, Austria. 11–13 July 2016

**DOI:** 10.1186/s13071-016-1902-x

**Published:** 2017-01-11

**Authors:** F. Simón, V. Kartashev, J. González-Miguel, A. Rivera, A. Diosdado, P. J. Gómez, R. Morchón, M. Siles-Lucas, Vladimir Kartashev, Nikolay Bastrikov, Boris Ilyasov, Alexey Ermakov, Sergey Kartashov, Denis Dontsov, Yuri Ambalov, Tamara Pavlikovska, Olga Sagach, Svetlana Nikolaenko, Nina Chizh, Alla Korzan, Alena Salauyova, Javier González-Miguel, Rodrigo Morchón, Mar Siles-Lucas, Fernando Simon, Éva Fok, István Kucsera, Sarah S. Übleis, Claudia Cuk, Michaela Nawratil, Victoria Wimmer, Carina Zittra, Julia Butter, Adelheid Obwaller, Karin Lebl, Thomas Zechmeister, Stefan Weiss, Georg G. Duscher, Herbert Auer, Anja Joachim, Hans-Peter Fuehrer, Sara Savic, Dubravka Pudar, Dusan Petric, Gioia Capelli, Fabrizio Montarsi, Cornelia Silaghi, Laura Kramer, Elena Carretón, Laura Peña, Sara Caceres, Gema Silvan, Juan Carlos Illera, José Alberto Montoya-Alonso, Esra Yilmaz, Moritz Fritzenwanker, Nikola Pantchev, Mathias Lendner, Sirichit Wongkamchai, Domenico Otranto, Inge Kroidl, Martin Dennebaum, Sabrina Ramünke, Roland Schaper, Georg von Samson-Himmelstjerna, Sven Poppert, Jürgen Krücken, Cristian-Ionut C. N. Florea, Poliana Gh Tudor, Stefan P. Olaru, Anca M. Dobrica, Artur Dobrzyński, Maciej Klockiewicz, Magdalena Wysmołek, Michał Czopowicz, Marta Parzeniecka-Jaworska, Joanna Nowakowska, Ewa Długosz, Anastasia Diakou, Mathios Mylonakis, Zoe Polizopoulou, Christos Koutinas, Simone Manzocchi, Stefano Di Palma, Martina Peloso, Nikola Pantchev, Nenad Milojković, Momčilo Aranđelović, Ljubomir Ćurčin, Barbora Mitková, Marcela Novotná, Jana Juránková, Lada Hofmannová, Dwight D. Bowman, David Modrý, Michael Leschnik, Ana Margarida Alho, Helder C. E. Cortes, Ana Patrícia Lopes, Maria João Vila-Viçosa, Luís Cardoso, Silvana Belo, Luís Madeira de Carvalho, Ana Margarida Alho, Hugo Vilhena, Ana Cristina Oliveira, Sara Granada, Ana Patrícia Lopes, Silvana Belo, Luís Madeira de Carvalho, Luís Cardoso, Radu Blaga, Virginie Daniel-Lesnard, Bruno Polack, Stéphanie Beurlet, Coralie Martin, Jacques Guillot, Lavinia Ciuca, Rodrigo Morchón, Ruxandra V. Moroti, Mihaela Arbune, Loredana Hurjui, Roman Constantin, Dumitru Acatrinei, Liviu Miron, Laura Kramer, Laura Rinaldi, Fernando Simón, Ewa Długosz, Agnieszka Szmidt, Artur Dobrzyński, Magdalena Wysmołek, Maciej Klockiewicz, Aleksandar M. Džamić, Tanja Kalezić, Ivana Čolović Čalovski, Dejan Rašić, Milan Cvetković, Sanja Mitrović, Rodrigo Morchón, Elena Carretón, Paula Josefina Gómez, Alicia Diosdado, Javier González-Miguel, Alicia Diosdado, Javier González-Miguel, Fernando Simón, Rodrigo Morchón, Vladan Panic, Rastko Bekvalac, Ivan Fenjac, Aleksandar Potkonjak, Suzana Otasevic, Sara Savic, Elias Papadopoulos, Athanasios Angelou, Eleftherios Gallidis, Kyriakos Spanoudis, Roland Schaper, Ramaswamy Chandrashekar, Ljubica Spasojevic Kosic, Vesna Lalosevic, Aleksandar Naglic, Stanislav Simin, Ljiljana Kuruca, Aleksandar Spasovic, Tomczuk Krzysztof, Szczepaniak Klaudiusz, Grzybek Maciek, Andrzej Junkuszew, Paulina Dudko, Pantchev Nikola, Stefaniak Marzena, Iwanicki Ryszard, Victoria Wimmer, Angela Monica Ionică, Carina Zittra, Natascha Leitner, Jan Votýpka, David Modrý, Andrei Daniel Mihalca, Hans-Peter Fuehrer, Manuela Schnyder, Malin Lange, Felipe Penagos, Carlos Hermosilla, Roland Schaper, Anja Taubert, Giulio Grandi, Eva Osterman-Lind, Roland Schaper, Ulrika Forshell, Manuela Schnyder, Viktória Čabanová, Zuzana Hurníková, Martina Miterpáková, Gary Conboy, Nicole Murphy, Tamara Hofstede, Dieter Barutzki, Viktor Dyachenko, Roland Schaper, Laetitia Lempereur, Ludovic Martinelle, Calixte Bayrou, Françoise Marechal, Anne-Catherine Dalemans, Bertrand J. Losson, Hany M. Elsheikha, Sarah B. Holmes, Nina Gillis-Germitsch, Manuela Schnyder, Gary Conboy, Nicole Guselle, Roland Schaper, Anastasia Diakou, Despina Migli, Angela Di Cesare, Dimitra Psalla, Dionisios Youlatos, Donato Traversa, Călin M. Gherman, Georgiana Deak, Angela M. Ionică, Gianluca D’Amico, Domenico Otranto, Andrei D. Mihalca, Malin Lange, Felipe Penagos, Tamara Muñoz-Caro, Gerd Magdowski, Uwe Gärtner, Helena Mejer, Roland Schaper, Carlos Hermosilla, Anja Taubert, Klaudiusz Szczepaniak, Krzysztof Tomczuk, Maciej Grzybek, Ryszard Iwanicki, Benjamin Bedel, Radu Blaga, Vassiliki Gouni, Valérie Chetboul, Ghita Benchekroun, Stéphane Blot, Patrick Verwaerde, Bruno Polack, Alice P. Hansen, Lene M. Vinther, Line K. Skarbye, Caroline S. Olsen, Helena Mejer, Jakob L. Willesen, Angela Di Cesare, Luigi Venco, Simone Manzocchi, Eleonora Grillotti, Edoardo Auriemma, Fabrizio Pampurini, Cecilia Garofani, Fabrizio Ibba, Donato Traversa, Felipe Penagos, Jesed Gutiérrez, Juan D. Velez, Diego Piedrahita, Malin Lange, Carlos Hermosilla, Anja Taubert, Jenny Chaparro, Fabio Macchioni, Marta Magi, Elisa Ulivieri, Francesca Gori, Manuela Schnyder

**Affiliations:** 1 Laboratory of Parasitology, Faculty of Pharmacy, University of Salamanca, Salamanca, 37007 Spain; 2 Rostov State Medical University, Rostov-na-Donu, 344022 Russia; 3 North Caucasus Research Veterinary Institute, Novocherkassk, 346421 Russia; 4 Laboratory of Parasitology, IRNASA, CSIC, Salamanca, 37008 Spain; 5 Rostov State Medical University, Rostov-na-Donu, 344022 Russia; 6 Rostov Oblast Diagnostic Center, Rostov-na-Donu, 344010 Russia; 7 North Caucasus Research Veterinary Institute, Novocherkassk, 346421 Russia; 8 Central Sanitary and Epidemiological Station of the Ukrainian Ministry of Health, Kiev, 01001 Ukraine; 9 Central Sanitary and Epidemiological Station of the Belorussian Ministry of Health, Minsk, 220000 Belarus; 10 Laboratory of Parasitology and IBSAL, University of Salamanca, Salamanca, 37007 Spain; 11 Instituto de Recursos Naturales y Agrobiología de Salamanca, CSIC, Salamanca, 37008 Spain; 12 Department of Parasitology and Zoology, University of Veterinary Science, István utca 2., H-1078 Budapest, Hungary; 13 Department of Parasitology, National Center for Epidemiology, Albert Flórián út 2-6., H-1097 Budapest, Hungary; 14 Department of Pathobiology, Institute of Parasitology, University of Veterinary Medicine Vienna, 1210 Vienna, Austria; 15 Federal Ministry of Defence and Sports, Division of Science, Research and Development, Vienna, Austria; 16 Institute for Veterinary Public Health, University of Veterinary Medicine Vienna, Vienna, Austria; 17 Biological Station Lake Neusiedl, Burgenland, Austria; 18 Schwabenberg 29, 8291 Burgauberg, Burgenland, Austria; 19 Institute of Specific Prophylaxis and Tropical Medicine, Medical University Vienna, A-1090 Vienna, Austria; 20 Scientific Veterinary Institute “Novi Sad, Novi Sad, Serbia; 21 Laboratory for Medical and Veterinary Entomology, Faculty of Agriculture, University of Novi Sad, Novie Sad, Serbia; 22 Laboratory of Parassitology Istituto Zooprofilattico Sperimentale delle Venezie, Legnaro, Italy; 23 National Centre for Vector Entomology, Vetsuisse Faculty, University of Zurich, Zurich, Switzerland; 24 Department of Veterinary Sciences, University of Parma, Parma, 43126 Italy; 25 Research Institute of Biomedical and Health Sciences (IUIBS), University of Las Palmas de Gran Canaria, 35001 Las Palmas, Spain; 26 Department of Veterinary Pathology, Complutense University of Madrid, 28040 Madrid, Spain; 27 Department of Animal Physiology, Complutense University of Madrid, 28040 Madrid, Spain; 28 Institute for Parasitology and Tropical Veterinary Medicine, Freie Universität Berlin, Berlin, Germany; 29 Institute of Medical Microbiology, Justus-Liebig-University, Giessen, Germany; 30 German Center for Infection Research (DZIF), Partner site Giessen-Marburg-Langen, Campus Giessen, Giessen, Germany; 31 IDEXX Laboratories, Ludwigsburg, Germany; 32 Institut für Parasitologie, Universität Leipzig, Leipzig, Germany; 33 Department of Parasitology, Faculty of Medicine Siriraj Hospital, Mahidol University, Bangkok, Thailand; 34 Department of Veterinary Medicine, University of Bari, 70010 Bari, Italy; 35 Division of Infectious Diseases and Tropical Medicine, Medical Centre of the University of Munich (LMU); German Center for Infection Research (DZIF), Partner site Munich, Munich, Germany; 36 Section Clinical Tropical Medicine, Department of Infectious Diseases, Heidelberg University Hospital, Heidelberg, Germany; 37 Bayer Animal Health GmbH, 40789 Monheim, Germany; 38 University Medical Center, Hamburg-Eppendorf, Germany; 39 Department of Parasitology and Parasitic Diseases, Faculty of Veterinary Medicine, University of Agronomical Sciences and Veterinary Medicine, Bucharest, 050097 Romania; 40 Praxis Vetlife, Bucharest, 021374 Romania; 41 Division of Parasitology and Invasiology, Faculty of Veterinary Medicine, Warsaw University of Life Sciences - SGGW, Ciszewskiego St. 8, 02-786 Warsaw, Poland; 42 Department of Small Animal Diseases with Clinic, Faculty of Veterinary Medicine, Warsaw University of Life Sciences - SGGW, Ciszewskiego St. 8, 02-786 Warsaw, Poland; 43 Laboratory of Veterinary Epidemiology and Economics, Faculty of Veterinary Medicine, Warsaw University of Life Sciences, Ciszewskiego St. 8, 02-786 Warsaw, Poland; 44 Department of Pathology and Veterinary Diagnostics, Faculty of Veterinary Medicine, Warsaw University of Life Sciences - SGGW, Ciszewskiego St. 8, 02-786 Warsaw, Poland; 45 Bayer Animal Health, Aleje Jerozolimskie 158, Warsaw, Poland; 46 Laboratory of Parasitology and Parasitic Diseases, School of Veterinary Medicine, Faculty of Health Sciences, Aristotle University of Thessaloniki, Thessaloniki, 54124 Greece; 47 Companion Animal Clinic, School of Veterinary Medicine, Faculty of Health Sciences, Aristotle University of Thessaloniki, Thessaloniki, 546 27 Greece; 48 Diagnostic Laboratory, School of Veterinary Medicine, Faculty of Health Sciences, Aristotle University of Thessaloniki, Thessaloniki, 546 27 Greece; 49 Novara Day Lab, IDEXX Laboratories, SP 9, Granozzo con Monticello (NO), 28060 Italy; 50 Animal Health Trust, Lanwades Park, Kentford, Newmarket, Suffolk CB8 7UU UK; 51 Ambulatorio Veterinario, Via Terraglio 194, Preganzion, 31022 Treviso, Italy; 52 IDEXX Laboratories, Mörikestr. 28/3, D-71636 Ludwigsburg, Germany; 53 Veterinary Clinic “Vet Centar”, 11000 Belgrade, Serbia; 54 Veterinary Clinic “Vet Alfa”, 11000 Belgrade, Serbia; 55 Veterinary Clinic “Intervet”, 11000 Belgrade, Serbia; 56 Department of Pathology and Parasitology, University of Veterinary and Pharmaceutical Sciences, Brno, 61242 Czech Republic; 57 CEITEC VFU, University of Veterinary and Pharmaceutical Sciences, Brno, 61242 Czech Republic; 58 Department of Microbiology & Immunology, College of Veterinary Medicine Cornell University, Ithaca, NY 14853 USA; 59 Biology Centre, Institute of Parasitology, Czech Academy of Sciences, České Budějovice, 370 05 Czech Republic; 60 Department for Companion Animals and Horses Veterinary University Vienna, Vienna, 1210 Austria; 61 CIISA, Faculty of Veterinary Medicine, ULisboa, Lisboa, Portugal; 62 Victor Caeiro Laboratory of Parasitology, Instituto de Ciências Agrárias e Ambientais Mediterrânicas (ICAAM), University of Évora, Évora, Portugal; 63 Department of Veterinary Sciences, School of Agrarian and Veterinary Sciences, University of Trás-os-Montes e Alto Douro (UTAD), Vila Real, Portugal; 64 Animal and Veterinary Research Centre (CECAV), UTAD, Vila Real, Portugal; 65 Medical Parasitology Unit, Global Health and Tropical Medicine, Instituto de Higiene e Medicina Tropical, Universidade Nova de Lisboa, Lisboa, Portugal; 66 CIISA, Faculty of Veterinary Medicine, ULisboa, Lisboa, Portugal; 67 Animal and Veterinary Research Centre (CECAV), University of Trás-os-Montes e Alto Douro (UTAD), Vila Real, Portugal; 68 Department of Veterinary Medicine, Escola Universitária Vasco da Gama, Coimbra, Portugal; 69 Hospital Veterinário do Baixo Vouga, Águeda, Portugal; 70 Clínica Casa dos Animais, Luanda, Angola; 71 Department of Veterinary Sciences, School of Agrarian and Veterinary Sciences, UTAD, Vila Real, Portugal; 72 Medical Parasitology Unit, Global Health and Tropical Medicine, Instituto de Higiene e Medicina Tropical, Universidade Nova de Lisboa, Lisboa, Portugal; 73 Parasitology department, BioPôle d’Alfort, Ecole nationale vétérinaire d’Alfort, UPE, Maisons-Alfort, France; 74 Clinique vétérinaire du Chêne Rouge, Chanteloup-en-Brie, France; 75 Vebio laboratory, Arcueil, France; 76 Muséum national d’Histoire naturelle, Paris, France; 77 Ion Ionescu de la Brad University of Agricultural Sciences and Veterinary Medicine Iasi, Iași, 700490 Romania; 78 Laboratory of Parasitology, University of Salamanca, Salamanca, 37007 Spain; 79 Department of Veterinary Medicine and Animal Productions, University of Naples Federico II, Naples, 80138 Italy; 80 Department of Veterinary Medicine, University of Parma, Parma, 43124 Italy; 81 Carol Davila University of Medicine and Pharmacy, Bucarest, 050474 Romania; 82 Faculty of Medicine and Pharmacy, Dunărea de Jos University of Galaţi, Galați, 800001 Romania; 83 Grigore T. Popa University of Medicine and Pharmacy, Iași, 700115 Romania; 84 Division of Parasitology and Invasiology, Faculty of Veterinary Medicine, Warsaw University of Life Sciences – SGGW, Ciszewskiego St. 8, 02-786 Warsaw, Poland; 85 Out-patients veterinary clinic “Przy Forcie”, Obrońców Tobruku St. 27 lok. 4, 01-494 Warsaw, Poland; 86 Department of Small Animal Diseases with Clinic, Faculty of Veterinary Medicine, Warsaw University of Life Sciences – SGGW, Nowoursynowska St. 159, 02-786 Warsaw, Poland; 87 Laboratory of Parasitology-Mycology, Institute of Microbiology and Immunology, University of Belgrade Faculty of Medicine, 11 000 Belgrade, Serbia; 88 Clinic for Eye Diseases, Clinical Center of Serbia, University of Belgrade Faculty of Medicine, 11 000 Belgrade, Serbia; 89 Laboratory of Parasitology, University of Salamanca, Salamanca, 37007 Spain; 90 Internal Medicine, University of Las Palmas de Gran Canaria, Gran Canaria, 35413 Spain; 91 Laboratory of Parasitology, University of Salamanca, Salamanca, 37007 Spain; 92 Private veterinary practice “Pedigre”, Novi Sad, Serbia; 93 Department of Veterinary medicine, Faculty of Agriculture, University of Novi Sad, Novi Sad, Serbia; 94 Department of Microbiology and Immunology, Medical faculty, University of Nis, Public Health Institute Nis, Nis, Serbia; 95 Scientific Veterinary Institute “Novi Sad, Novi Sad, Serbia; 96 School of Veterinary Medicine, Aristotle University, Thessaloniki, 541 24 Greece; 97 Bayer Animal Health GmbH, Leverkusen, 51368 Germany; 98 IDEXX Laboratories, Inc., Westbrook, ME 04092 USA; 99 Department of Veterinary Medicine, Faculty of Agriculture, University of Novi Sad, Novi Sad, 21000 Serbia; 100 JKP “Zoohigijena i veterina”, Novi Sad, 21000 Serbia; 101 PVA “Mama”, Belgrade, 11000 Serbia; 102 Department of Parasitology and Invasive Diseases, University of Life Sciences, Lublin, ul. Akademicka 12, 20-950 Lublin, Poland; 103 Faculty of Biology and Animal Breeding Department of Small Ruminants Breeding and Agriculture Advisory, University of Life Sciences, In Lublin ul. Akademicka 13, 20-950 Lublin, Poland; 104 IDEXX Laboratories, 71636 Ludwigsburg, Germany; 105 Lubelskie Centrum Małych Zwierząt, ul. Stefczyka 11, 20-151 Lublin, Poland; 106 Department of Pathobiology, Institute of Parasitology, University of Veterinary Medicine Vienna, 1210 Vienna, Austria; 107 Department of Parasitology and Parasitic Diseases, Faculty of Veterinary Medicine, University of Agricultural Sciences and Veterinary Medicine, Calea Mănăştur 3-5, Cluj-Napoca, Romania; 108 Department of Parasitology, Faculty of Sciences, Charles University, Viničná 7, 12844 Prague, Czech Republic; 109 Department of Pathology and Parasitology, Faculty of Veterinary Medicine, University of Veterinary and Pharmaceutical Sciences, Palackého tr. 1946/1, 612 42 Brno, Czech Republic; 110 Biology Centre, Institute of Parasitology, Czech Academy of Sciences, Branisovska 31, 370 05 České Budějovice, Czech Republic; 111 Institute of Parasitology, University of Zurich, 8057 Zurich, Switzerland; 112 Institute of Parasitology, Justus Liebig University, Gießen, 35392 Germany; 113 Bayer Health GmbH, Leverkusen, 51368 Germany; 114 Department of Microbiology, National Veterinary Institute, Uppsala, 75189 Sweden; 115 Bayer Animal Health GmbH, Leverkusen, Germany; 116 Bayer HealthCare - Animal Health, Copenhagen, Denmark; 117 Institute of Parasitology – University of Zurich, Zurich, Switzerland; 118 Institute of Parasitology, Slovak Academy of Sciences, Košice, 040 01 Slovakia; 119 Department of Pathology and Microbiology, Atlantic Veterinary College, Charlottetown, Prince Edward Island C1A 4P3 Canada; 120 Animal Health Bayer Inc, Mississauga, Ontario L4W 5R6 Canada; 121 Veterinary Laboratory Freiburg, Freiburg, 79108 Germany; 122 Bayer Animal Health GmbH, Leverkusen, 51368 Germany; 123 University of Liège, Faculty of Veterinary Medicine, Laboratory of Parasitology and Parasitic Diseases, Liège, Belgium; 124 University of Liège, Faculty of Veterinary Medicine, Experimental Station CARE – FePex, Center for Fundamental and Applied Research for Animal and Health (FARAH), Liege, Belgium; 125 University of Liège, Faculty of Veterinary Medicine, Laboratory of Pathology, Liège, Belgium; 126 Bayer Health Care, Diegem, Belgium; 127 University of Nottingham, Loughborough, Leicestershire LE12 5RD UK; 128 Institute of Parasitology, Vetsuisse-Faculty, University of Zurich, Zürich, 8057 Switzerland; 129 Department of Pathology and Microbiology, Atlantic Veterinary College, Charlottetown, Prince Edward Island C1A 4P3 Canada; 130 Bayer Animal Health GmbH, Leverkusen, Germany; 131 Laboratory of Parasitology and Parasitic Diseases, School of Veterinary Medicine, Faculty of Health Sciences, Aristotle University of Thessaloniki, Thessaloniki, 54124 Greece; 132 Department of Zoology, School of Biology, Aristotle University of Thessaloniki, Thessaloniki, 54124 Greece; 133 Faculty of Veterinary Medicine, Teaching Veterinary Hospital, Località Piano d’Accio snc, Teramo, 64100 Italy; 134 Laboratory of Pathology, School of Veterinary Medicine, Faculty of Health Sciences, Aristotle University of Thessaloniki, Thessaloniki, 54124 Greece; 135 Department of Parasitology, University of Agricultural Sciences and Veterinary Medicine Cluj-Napoca, Calea Mănăștur 3-5, 400372 Cluj-Napoca, Romania; 136 Dipartimento di Medicina Veterinaria, Università degli Studi di Bari, Bari, Italy; 137 Institute of Parasitology, Justus Liebig University, Gießen, 35392 Germany; 138 Institute of Anatomy and Cell biology, Justus Liebig Universität, Giessen, Germany; 139 Parasitology and Aquatic Diseases, University of Copenhagen, 1871 Copenhagen, Denmark; 140 Bayer Health GmbH, Leverkusen, 51368 Germany; 141 Department of Parasitology and Invasive Diseases, University of Life Sciences, Lublin, ul. Akademicka 12, 20-950 Lublin, Poland; 142 Lubelskie Centrum Małych Zwierząt, ul. Stefczyka 11, 20-151 Lublin, Poland; 143 Department of Emergency and intensive care, Centre Hospitalier Universitaire, Université Paris-Est, Ecole Nationale Vétérinaire d’Alfort, 94704 Maisons-Alfort, France; 144 Department of parasitology, BioPôle, Université Paris-Est, Ecole Nationale Vétérinaire d’Alfort, 94704 Maisons-Alfort, France; 145 Department of Cardiology, Centre Hospitalier Universitaire, Université Paris-Est, Ecole Nationale Vétérinaire d’Alfort, 94704 Maisons-Alfort, France; 146 Department of Small Animal Internal Medicine, Centre Hospitalier Universitaire, Université Paris-Est, Ecole Nationale Vétérinaire d’Alfort, 94704 Maisons-Alfort, France; 147 Department of Veterinary Disease Biology, University of Copenhagen, 1870 Frb C, Denmark; 148 Environmental Support, DFP, Novo Nordisk, 4400 Kalundborg, Denmark; 149 Department of Veterinary Clinical and Animal Sciences, University of Copenhagen, 1870 Frb C., Denmark; 150 Faculty of Veterinary Medicine, University of Teramo, 64100 Teramo, Italy; 151 Veterinary Hospital “Città di Pavia”, Viale Cremona 179, 27100 Pavia, Italy; 152 Novara Day Lab - IDEXX Laboratories Italia, Granozzo con, Monticello Italy; 153 Veterinary Pratice “Centro Italia”, Via Biancifiori 3, 02100 Rieti, Italy; 154 Istituto Veterinario di Novara, S.P. 9, 28060 Granozzo con Monticello, Italy; 155 Bayer Sanità Animale, Viale Certosa 130, 20156 Milan, Italy; 156 Veterinary Pratice “Poggio dei Pini” Strada 40, 09012 Capoterra, Cagliari Italy; 157 CIBAV research group, Veterinary Medicine School, Faculty of Agrarian Sciences, University of Antioquia, Medellín, Antioquia 050034 Colombia; 158 Institute of Parasitology, Justus Liebig University Giessen, Giessen, 35392 Germany; 159 Department of Veterinary Science, University of Pisa, Viale delle Piagge 2, 56124 Pisa, Italy; 160 Institute of Parasitology, Vetsuisse Faculty, University of Zurich, 8057 Zurich, Switzerland

## TOPIC 1: Dirofilarioses (Humans, Mosquitoes)

### A1 Human dirofilariosis in Europe: basic facts and retrospective review

#### F Simón^1^, V Kartashev^2,3^, J González-Miguel^1^, A Rivera^1^, A Diosdado^1^, PJ Gómez^1^, R Morchón^1^, M Siles-Lucas^4^

##### ^1^Laboratory of Parasitology, Faculty of Pharmacy, University of Salamanca, Salamanca, 37007, Spain; ^2^Rostov State Medical University, Rostov-na-Donu, 344022, Russia; ^3^North Caucasus Research Veterinary Institute, Novocherkassk, 346421, Russia; ^4^Laboratory of Parasitology, IRNASA, CSIC, Salamanca, 37008, Spain

###### **Correspondence:** F Simón (fersimon@usal.es)

In Europe domestic and sylvatic canines and felines are the reservoirs of *Dirofilaria immitis* and *D. repens*, while different culicid mosquito species act as vectors of these species. Many mosquito species feed indiscriminately on animal reservoirs and man, thus where there is canine dirofilariosis, the risk of zoonotic infections exists. There are three forms of human dirofilariosis: Pulmonary dirofilariosis (PD), usually causing a solitary pulmonary nodule attributed to *D. immitis*; subcutaneous dirofilariosis (SD) manifesting as subcutaneous nodules located in different parts of the body and ocular dirofilariosis (OD) in which worms cause nodules or remain unencapsulated in the eye area, being the last two variants mainly caused by *D. repens*. Most of the information on human dirofilariosis is generated by the clinical cases reported and their retrospective review, but there is very scarce other kind of studies. In Europe continues the sharp increase of SD/OD cases unlike the extremely low number of reports of PD cases, without being able to indicate the objective causes of this fact, since both species are present in animal reservoirs of the continent. Most of these cases have been reported in Ukraine and the Russian Federation [1], although a significant number were detected in recent years in Belarus, Balkan and central European countries. The increase in case reports revealed new locations and clinical implications, which are forcing to reassess the prognosis and severity of many cases. Molecular techniques established that worms of *D. repens* with ocular localization are genetically identical to those located in the subcutaneous tissue and the participation of *D. immitis* in OD in Ukraine, where this species seems to be the causal agent of the ocular variant in the 13.8% of cases. The routine application of non-invasive techniques such as ultrasound and Doppler helps to establish a rapid prognosis and diagnosis, consistent with the non-malignant nature of nodules in both SD and OD. Studies using "in vitro" cultures of vascular endothelial and smooth muscle cells have demonstrated the ability of some *Dirofilaria* molecules to activate the fibrinolytic system and enhance the generation of plasmin. Plasmin plays a dual role contributing to remove thrombi, but also participating in the stimulation of mechanisms leading to villous endarteritis, such as cell proliferation and migration [2]. Although not specifically focused on human dirofilariosis, these studies can contribute to a deeper understanding of the pathophysiology of human dirofilariosis.

**References**

1. Kartashev V, Tverdokhlebova T, Korzan A, Vedenkov A, Simón L, González-Miguel J, Morchón R, Siles-Lucas M, Simón F. Human subcutaneous/ocular dirofilariosis in Russian Federation and Belarus, 1997-2013. *Internat. J. Infect. Dis.*, 2015, 33:209–11.

2. González-Miguel J, Siles-Lucas M, Kartashev V, Simón F. Plasmin in parasitic chronic infections: Friend or foe? *Trends Parasitol.*, 2016, 32(4):325–35.

### A2 Human dirofilariasis – morbidity, clinical presentation, and diagnosis

#### Vladimir Kartashev^1,3^, Nikolay Bastrikov^1^, Boris Ilyasov^2^, Alexey Ermakov^3^, Sergey Kartashov^3^, Denis Dontsov^1^, Yuri Ambalov^1^, Tamara Pavlikovska^4^, Olga Sagach^4^, Svetlana Nikolaenko^4^, Nina Chizh^4^, Alla Korzan^5^, Alena Salauyova^5^, Javier González-Miguel^6^, Rodrigo Morchón^6^, Mar Siles-Lucas^7^, Fernando Simon^6^

##### ^1^Rostov State Medical University, Rostov-na-Donu, 344022, Russia; ^2^Rostov Oblast Diagnostic Center, Rostov-na-Donu, 344010, Russia; ^3^North Caucasus Research Veterinary Institute, Novocherkassk, 346421, Russia; ^4^Central Sanitary and Epidemiological Station of the Ukrainian Ministry of Health, Kiev, 01001, Ukraine; ^5^Central Sanitary and Epidemiological Station of the Belorussian Ministry of Health, Minsk, 220000, Belarus; ^6^Laboratory of Parasitology and IBSAL, University of Salamanca, Salamanca, 37007, Spain; ^7^Instituto de Recursos Naturales y Agrobiología de Salamanca, CSIC, Salamanca, 37008, Spain

###### **Correspondence:** Vladimir Kartashev (vkrt@yandex.ru)

As many as 3,545 cases of human dirofilariasis were recognized in Russia, Ukraine, and Belorussia starting from 1997. Clinical problems of human dirofilariasis become an issue and need be thoroughly analyzed. A patient self-assessment, the parasite anatomical location and clinical manifestation determine diagnostic workup. Five patients with peritonitis were operated immediately and *Dirofilaria* was unexpectedly found in peritoneal cavity. In contrast – five patients with “silent” pulmonary dirofilariasis were diagnosed late and accidentally. Affected eye (37% of all patients, variations 22 – 48% in different years) in the case of a foreign “moving entity” in an eye or eyelid conjunctiva (19%) or with eye acute inflammation (25%) strongly motivated a patient to visit a doctor in the contrast with patients with slowly growing “silent” nodule (56%). Anyway as many as 86% of the patients with eye located *Dirofilaria* were addressed to a doctor during the first month of the disease. Nearly equal proportion of patients (around 62%) with head (28%), or trunk (12%), or man’s genitalia (3%) located parasite also visited a doctor in the first month of the disease. Female patients with breast location (3%) were consulted earlier and were undergone surgery in short time (in the first 2 weeks) mostly because the main diagnostic hypothesis was breast cancer. In the cases of extremities located parasitic nodule (hands – 9.4%, legs – 8.6%) only 31% and 36% of patients (accordingly) decided to be consulted by a doctor in the first month from the onset because they did not regard their condition as life-threatening. The next important issue is doctor information about dirofilariasis, his specialty and previous experience. Everything had great influence on timely and correct diagnosis or at least on inclusion of dirofilariasis in the list of diagnostic hypotheses. In the territories with sporadic morbidity only few doctors (7%) suspected dirofilariasis before surgery – they mostly diagnosed benign or malignant tumors (72%). There is a contrast with endemic territories where dirofilariasis was suspected by doctors in the much higher proportion of the patients (85%). Preliminary ultrasound and color Doppler examination of patients with dirofilariasis made great input in the diagnosis. The findings include hypoechoic encapsulated linear structures without internal blood vessels and sometimes (47%) with detectable movement of the parasite. Those findings allowed to exclude malignancies before surgery in all ultrasound examined patients. Medical community has to be better informed about dirofilariasis. Ultrasound should be a standard procedure in patients with subcutaneous nodules.

### A3 A few thoughts about the recent epidemiological situation of dirofilariosis in Hungary with particular regard to quick spread and high prevalences in certain areas

#### Éva Fok^1^, István Kucsera^2^

##### ^1^Department of Parasitology and Zoology, University of Veterinary Science, István utca 2., H-1078 Budapest, Hungary; ^2^Department of Parasitology, National Center for Epidemiology, Albert Flórián út 2-6., H-1097 Budapest, Hungary

###### **Correspondence:** Éva Fok (hanavica.fok13@gmail.com)

Dirofilariosis is an emerging zoonosis in Hungary. The first autochthonous *Dirofilaria repens* infection of dogs were diagnosed in the end of the 90’s, then soon in 2007 the first dog infected with *D. immitis* was detected and in 2010 a pet ferret case was published, too. A first comprehensive countrywide survey showed that most of *D. repens* infected dog cases (prevalence: 18-46%) occurred in the eastern part of Hungary, namely on the Great Hungarian Plain along the Tisza river and its branches [1]. The findings of this earlier study were partly confirmed by later surveys [2, 3, 5], but these studies mainly focused on the heartworm incidence. It is stated [5] that the climate of the Great Hungarian Plain is the most suitable region for the establishment of *D. immitis* in Hungary. Although sporadic cases in wild canines (such as foxes and jackals) and domestic dogs also occur in other regions of the country it is slightly worrying that the main habitat of *D. immitis* might be in Szeged town or in the Southern Great Hungarian Plain. This assumption may strengthen by earlier (unpublished) and newer necropsy records [5]. Moreover the first molecular screening of the vector mosquitoes collected in Szeged revealed that not only *D. immitis* was present in the specimens but also DNA of *D. repens* [4]. So far, in Hungary human dirofilariosis is caused by *D. repens*. Since the first reported human case, 115 further episodes were diagnosed in Hungary [6]. Evaluation of the territorial distribution of human episodes revealed that most infections occurred in patients living in the Danube-Tisza interflow region and eastern part of the country. The spread of the “greenhouse effect” lead to the extension of the Mediterranean climatic belt to the north giving better opportunities for both vectors and worms to thrive and spawn infection. A close cooperation not only with the parasitologists, but also between practicing veterinarians and medical doctors is necessary to organise the control against both *Dirofilaria* species.

**References**

1. Jacsó O. Prevalence of *Dirofilaria* spp. in Hungary and veterinary importance, the experience of treatment. PhD thesis, 2014. http://www.huveta.hu/bitstream/handle/10832/1024/Jacso%20Olga%20Thesis%20English.pdf?sequence=2

2. Farkas R., Gyurkovszky M., Lukács Z., Aladics B., Solymosi N.: Seroprevalence of some vector-borne infections of dogs in Hungary. Vector Borne Zoonotic Dis., 2014; 14(4):256–260.

3. Tolnai Z, Széll Z, Sproch Á, Szeredi L, Sréter T. *Dirofilaria immitis*: An emerging parasite in dogs, red foxes and golden jackals in Hungary. Vet Parasitol., 2014; 203 (3-4):339–342.

4. Zittra C., Kocziha Zs., Pinnyei Sz., Harl J., Kieser K., Laciny A., Barbara Eigner B., Silbermayr K., Duscher G. G., Éva Fok É., Fuehrer H.-P. Screening blood-fed mosquitoes for the diagnosis of filarioid helminths and avian malaria. Parasites & Vectors, 2015; 8:16.

5. Bacsadi Á., Papp, A., Szeredi L., Tóth, G., Nemes, C., Imre, V., Tolnai, Z., Széll, Z., Sréter T. Retrospective study on the distribution of *Dirofilaria immitis* in dogs in Hungary. Vet Parasitol., 2016; 220:83–86.

6. Dóczi I., Bereczki L., Gyetvai T., Fejes I., Skribek Á., Szabó Á., Berkes Sz., Tiszlavicz L., Bartha N., Bende B., Kis E., Kucsera I. Description of five dirofilariosis cases in South Hungary and review epidemiology of this disease for the country. Wien. Klin. Wochenschr, 2015; 127:696–702.

### A4 An update on the current situation of *Dirofilaria repens* and *Dirofilaria immitis* in Austria

#### Sarah S. Übleis^1^, Claudia Cuk^1^, Michaela Nawratil^1^, Victoria Wimmer^1^, Carina Zittra^1^, Julia Butter^1^, Adelheid Obwaller^2^, Karin Lebl^3^, Thomas Zechmeister^4^, Stefan Weiss^5^, Georg G. Duscher^1^, Herbert Auer^6^, Anja Joachim^1^, Hans-Peter Fuehrer^1^

##### ^1^Department of Pathobiology, Institute of Parasitology, University of Veterinary Medicine Vienna, 1210 Vienna, Austria; ^2^Federal Ministry of Defence and Sports, Division of Science, Research and Development, Vienna, Austria; ^3^Institute for Veterinary Public Health, University of Veterinary Medicine Vienna, Vienna, Austria; ^4^Biological Station Lake Neusiedl, Burgenland, Austria; ^5^Schwabenberg 29, 8291 Burgauberg, Burgenland, Austria; ^6^Institute of Specific Prophylaxis and Tropical Medicine, Medical University Vienna, A-1090 Vienna, Austria

###### **Correspondence:** Hans-Peter Fuehrer (hans-peter.fuehrer@vetmeduni.ac.at)

*Dirofilaria immitis* and *D. repens* are filarioid helminths with domestic and wild canids as main hosts and mosquitoes as vectors. Both species are known to be zoonotic. *Dirofilaria repens* and *D. immitis* seem associated with climate change and a spread from historically endemic countries in Southern Europe to Central Europa was observed. Until very recently both species were known not to be endemic in Austria [1]. In Austria most cases of *Dirofilaria* spp. in humans and dogs are introduced. However, rarely infections with *D. repens* were discussed to be autochthonous. The introduction of *D. repens* to Austria was confirmed within a mosquito surveillance in Burgenland (Eastern Austria) for the first time in its vector [2,3,4]. We summarize not only introduced and possible autochthonous cases of *Dirofilaria* sp. in humans, dogs and vectors in Austria, but will also present data of mosquito screenings conducted after the first findings of *D. repens* in *Anopheles algeriensis* and the *An. maculipennis*-complex [3]. Moreover novel diagnostic tools for these filarioid helminths will be presented.

This study was funded by the ERA-Net BiodivERsA, with the national funders FWF I-1437, ANR-13-EBID-0007-01 and DFG BiodivERsA KL 2087/6-1 as part of the 2012-13 BiodivERsA call for research proposals.

**References**

1. Fuehrer HP, Auer H, Leschnik M, Silbermayr K, Duscher G, Joachim A. *Dirofilaria* in Humans, Dogs, and Vectors in Austria (1978-2014)-From Imported Pathogens to the Endemicity of *Dirofilaria repens*. PLoS Negl Trop Dis. 2016, 10 (5): e0004547. doi: 10.1371/journal.pntd.0004547.

2. Auer H, Susani M. The first autochthonous case of subcutaneous dirofilariosis in Austria. Wien Klin Wochenschr., 2008, 120 (19-20 Suppl 4):104-6.

3. Duscher G, Feiler A, Wille-Piazzai W, Bakonyi T, Leschnik M, Miterpáková M, Kolodziejek J, Nowotny N, Joachim A. Detection of *Dirofilaria* in Austrian dogs. Berl Munch Tierarztl Wochenschr., 2009, 122 (5-6): 199–203.

4. Silbermayr K, Eigner B, Joachim A, Duscher GG, Seidel B, Allerberger F, Indra A, Hufnagl P, Fuehrer HP. Autochthonous *Dirofilaria repens* in Austria. Parasit Vectors., 2014, 7: 226. doi: 10.1186/1756-3305-7-226.

### A5 Experimental assessment of vector competence of different mosquito species for *Dirofilaria immitis*

#### Sara Savic^1^, Dubravka Pudar^2^, Dusan Petric^2^, Gioia Capelli^3^, Fabrizio Montarsi^3^, Cornelia Silaghi^4^

##### ^1^Scientific Veterinary Institute “Novi Sad, Novi Sad, Serbia; ^2^Laboratory for Medical and Veterinary Entomology, Faculty of Agriculture, University of Novi Sad, Novie Sad, Serbia; ^3^Laboratory of Parassitology Istituto Zooprofilattico Sperimentale delle Venezie, Legnaro, Italy; ^4^National Centre for Vector Entomology, Vetsuisse Faculty, University of Zurich, Zurich, Switzerland

###### **Correspondence:** Sara Savic (sara@niv.ns.ac.rs)

Heart worm disease caused by *Dirofilaria immitis* is well known in Southern parts of Europe. In the past decade several studies have been performed on its diagnosis, treatment and prevention, but knowledge on vector competence of Central European mosquito species for *D. immitis* under local climate conditions is still scarce.

The aim of this study was therefore to analyze the vector competence of three different mosquito species (*Aedes vexans, Ae. geniculatus* and *Culex pipiens* biotype *molestus*, endemic in Serbia and Switzerland) for *D. immitis* at constant and realistic fluctuating temperature regimes under laboratory conditions.

Mosquitoes were kept in the laboratory at 24-27 °C, with 85% of humidity and fed with sugar solution. Six groups of female mosquitoes (30-52 individuals), were fed with blood containing *D. immitis* microfilariae (6,000 mf/ml) obtained from a naturally infected dog using artificial feeding methods (Hemotek, a blood sausage, cotton stick method). After inoculation, blood fed mosquitoes were incubated at constant or realistic fluctuating temperature of 27 °C (17.5-35 °C, with average 27 °C). Parameters determined were: feeding rate, infectious dose, mortality rate, infection rates and infectivity rates. Mosquitoes were dissected under a stereomicroscope on days 2, 4, 7, 10, 14 post inoculation (p.i.), to observe the developmental stages of the filariae. Additionally, PCR analysis was performed. The feeding rate ranged from 26% in *Cx pipiens* biotype *molestus* to 61-63% in *Ae.vexans* groups and to 74% in *Ae.geniculatus.* The observed infectious dose was 7-10 microfilaria in *Ae.vexans*. No live microfilariae could be observed in the other species in samples taken on day 1 p.i. Mortality rate was rather high, it was altogether, 78% during the 14 days of incubation period and ranged from 12.5% in *Cx.pipiens* biotype *molestus* fed with cotton stick to 100% in those fed with Hemotek. The highest percentage (73.9%) of mosquitoes died until day 4 p. i (feeding). In *Ae.geniculatus* and *Ae.vexans* groups, microfilariae were found until day 7 p.i., L1 from day 4-10p.i. and L2 were found only in *Ae.vexans* groups on day 10. In *Cx.pipens* biotype *molestus* no larval stages were found by microscopy. PCR analysis revealed the highest number of specimens positive for *Dirofilaria* in *Ae.vexans*, kept at constant 27 °C. All of the *Cx pipiens* biotype *molestus* mosquitoes that died during the experiment were PCR positive. Female mosquitoes developing infectious L3 stages (infectivity rates), L3 were identified only in *Ae. vexans* at day 14 p.i. Total infection rates (microscopy and PCR) were 72% for *Ae vexans* kept at constant 27 °C, 56.6% for *Ae vexans* kept at 27 °C fluctuating, 70% for *Ae. geniculatus* and 37.5% for *Cx pipiens* biotype *molestus* fed with the Hemotek. To conclude, vector competence for *D. immitis* was shown for the flood water mosquito *Ae. vexans* both at constant and fluctuating 27 °C. The respective results were not yet conclusive for *Cx pipiens* biotype *molestus* and *Ae.geniculatus* and further studies will be necessary with these species.

## TOPIC 2: Dirofilarioses (Endosymbionts and Molecular Biology/Veterinary Medicine)

### A6 Advances in adulticide treatment in canine heartworm disease (*Dirofilaria immitis*): Macroyclic lactones and doxycycline

#### Laura Kramer (kramerlh@unipr.it)

##### Department of Veterinary Sciences, University of Parma, Parma 43126 Italy

Currently, the only registered drug for adulticide therapy in dogs with heartworm disease (HWD; *Dirofilaria immitis*) is melarsomine dihydrochloride (Immiticide®, Merial). The American Heartworm Society (AHS), based on results from several studies [1, 2], currently recommends that, in cases where arsenical therapy is not possible or is contraindicated, a monthly heartworm preventive along with doxycycline for a 4-week period might be considered. There is no published data on the use of moxidectin in combination with doxycycline. Preliminary results of an on-going study* show that moxidectin, the only macrocyclic lactone (ML) registered as a microfilaricide, is also adulticidal when combined with doxycycline. Interestingly, a similar combination therapy has been shown to be highly effective against human body lice, an ectoparasite that has been shown to develop resistance to MLs and which also harbours bacterial endosymbionts [3]. It is not yet known if the efficacy of antibiotics and MLs is due to pharmacokinetic or pharmacodynamic synergism. It has been shown that compounds including antibiotics can increase intracellular concentrations of MLs and that MLs can inhibit cell detoxification mechanisms, thus increasing intracellular concentrations of drugs, including antibiotics [4]. A recent study has shown, however, that serum levels of doxycycline in dogs treated with the combination protocol were not statistically different compared to dogs treated with doxycycline alone [5]. It would be of interest, and a research priority, to elucidate the nature of this synergy.

This study was funded by University of Parma (65/OPBA/2015).

**References**

1. Grandi G, Quintavalla C, Mavropoulou A, et al. A combination of doxycycline and ivermectin is adulticidal in dogs with naturally acquired heartworm disease (*Dirofilaria immitis*). Vet Parasitol., 2010, 169 (3-4): 347–351.

2. Kramer L, Grandi G, Passeri B, et al. Evaluation of lung pathology in *Dirofilaria immitis*-experimentally infected dogs treated with doxycycline or a combination of doxycycline and ivermectin before administration of melarsomine dihydrochloride. Vet Parasitol., 2011, 176 (4): 357–360.

3. Sangaré AK, Rolain JM, Gaudart J, Weber P, Raoult D. Synergistic activity of antibiotics combined with ivermectin to kill body lice. Int J Antimicrob Agents., 2016, 47 (3): 217–223.

4. Guseman AJ, Miller K, Kunkle G, et al. Multi-Drug Resistance Transporters and a Mechanism-Based Strategy for Assessing Risks of Pesticide Combinations to Honey Bees. PLoS One. 2016,11 (2).

5. Menozzi A, Bertini S, Turin L, et al. Doxycycline levels and anti-*Wolbachia* antibodies in sera from dogs experimentally infected with *Dirofilaria immitis* and treated with a combination of ivermectin/doxycycline. Vet Parasitol. 2015, 209 (3): 281–284.

### A7 Cortisol as indicator of stress in heartworm infection in dogs

#### Elena Carretón^1^, Laura Peña^2^, Sara Caceres^3^, Gema Silvan^3^, Juan Carlos Illera^3^, José Alberto Montoya-Alonso^1^

##### ^1^ Research Institute of Biomedical and Health Sciences (IUIBS), University of Las Palmas de Gran Canaria, 35001 Las Palmas, Spain; ^2^Department of Veterinary Pathology, Complutense University of Madrid, 28040 Madrid, Spain; ^3^Department of Animal Physiology, Complutense University of Madrid, 28040 Madrid, Spain

###### **Correspondence:** Elena Carretón (elena.carreton@ulpgc.es)

Cortisol, a steroid produced in the adrenal cortex, is a key hormone involved in the stress response and serum levels have often been used as a measure of stress. It has been demonstrated that prolonged stress, as indicated by cortisol levels, is associated with reduced survival, fecundity, and immunity [1]. Studies have examined interactions between parasites and cortisol levels in some species, with discrepant results [2]. The aims of this study were to evaluate the potentially stressful effects of the infection of *Dirofilaria immitis* in dogs by measuring the levels of serum cortisol before and after the adulticide treatment.

Serums from 61 dogs positive to *D.immitis* antigens were included; all blood samples were further examined by the modified Knott test. The parasite burden was assessed by echocardiography in 51 of these dogs [3]. Furthermore, 22 dogs were treated following the AHS protocol and additional blood samples were taken on days 60, 90 and 120. Serum cortisol was measured by EIA Method, validated for this species. There were 24 females and 37 males. Thirty were client-owned dogs and 31 lived in a local shelter; 41 were microfilaremic and 20 were amicrofilaremic; 26 were symptomatic and 35 were asymptomatic. When the parasite burden was asses (n = 51), 20 had high burden and 31 had low burden. The mean level of cortisol in heartworm infected dogs was 10.08 ± 8.16 ng/ml. There were not statistically significant differences between sex and microfilaremic status, but there were between symptomatic and asymptomatic dogs (p < 0.05). When parasite burden was evaluated, dogs with high burden had significantly greater levels of cortisol (p < 0.001). During the adulticide treatment, the levels of cortisol dropped gradually in each sampling, being the cortisol levels from day 120 within the reference ranges (2.31 ± 1.02 ng/ml). Shelter versus client-owned dogs had higher cortisol levels (p < 0.05).

The results demonstrate presence of stress in dogs infected by *D.immitis*, especially in symptomatic dogs, and those with high parasite burden similar to a previous study [4]. These results are similar to other studies which evaluated the effect of several parasites in animals and humans; while the different results found in other research may be caused by the small virulence of the parasites studied [2]. On the other hand, as the parasites are being removed, the levels of cortisol gradually decreased. Although not the aim of the study, we could observe that dogs from shelter had higher levels of cortisol, consistent with previous studies [5].

**Trial registration/ Consent to publish**

The study was approved by the ethical committee of Veterinary Medicine Service of Las Palmas de Gran Canaria University (MV-2016/07) and was carried out in accordance with the current European legislation on animal protection.

**References**

1. Snaith TV, Chapman CA, Rothman JM, Wasserman MD. Bigger groups have fewer parasites and similar cortisol levels: a multi-group analysis in red colobus monkeys. Am J Primatol. 2008; 70: 1072–1080.

2. Monello RJ, Millspaugh JJ, Woods RJ, Gompper ME. The influence of parasites on faecal glucocorticoid metabolite levels in raccoons: an experimental assessment in a natural setting. J. Zool. 2010; 282: 100–108.

3. Venco, L., Genchi, C., Vigevani Colson, P., Kramer, L., 2003. Relative utility of echocardiography, radiography, serologic testing and microfilariae counts to predict adult worm burden in dogs naturally infected with heartworms. In: Seward, R.L., Knight, D.H. (Eds.), Recent Advances in Heartworm Disease, Symposium’01. American Heartworm Society, Batavia, IL, pp. 111–124.

4. Fleming MW. Cortisol as an Indicator of Severity of Parasitic Infections of Haemonchus contortus in Lambs (Ovis aries). Comp Biochem Physiol B Biochem Mol Biol. 1997; 116: 41–44.

5. Coppola CL, Grandin T, Enns RM. Human interaction and cortisol: can human contact reduce stress for shelter dogs? Physiol Behav. 2006; 87:537–541.

### A8 Mitochondrial genome sequences of the zoonotic canine filariae *Dirofilaria* (*Nochtiella*) *repens* and *Candidatus* Dirofilaria (Nochtiella) honkongensis

#### Esra Yilmaz^1^, Moritz Fritzenwanker^2^, Nikola Pantchev^3^, Mathias Lendner^4^, Sirichit Wongkamchai^5^, Domenico Otranto^6^, Inge Kroidl^7^, Martin Dennebaum^8^, Sabrina Ramünke^1^, Roland Schaper^9^, Georg von Samson-Himmelstjerna^1^, Sven Poppert^10^, Jürgen Krücken^1^

##### ^1^Institute for Parasitology and Tropical Veterinary Medicine, Freie Universität Berlin, Berlin, Germany; ^2^Institute of Medical Microbiology, Justus-Liebig-University, Giessen, Germany; German Center for Infection Research (DZIF), Partner site Giessen-Marburg-Langen, Campus Giessen, Giessen, Germany; ^3^IDEXX Laboratories, Ludwigsburg, Germany; ^4^Institut für Parasitologie, Universität Leipzig, Leipzig, Germany; ^5^Department of Parasitology, Faculty of Medicine Siriraj Hospital, Mahidol University, Bangkok, Thailand; ^6^Department of Veterinary Medicine, University of Bari, 70010, Bari, Italy; ^7^Division of Infectious Diseases and Tropical Medicine, Medical Centre of the University of Munich (LMU); German Center for Infection Research (DZIF), Partner site Munich, Munich, Germany; ^8^Section Clinical Tropical Medicine, Department of Infectious Diseases, Heidelberg University Hospital, Heidelberg, Germany; ^9^Bayer Animal Health GmbH, 40789, Monheim, Germany; ^10^University Medical Center, Hamburg-Eppendorf, Germany

###### **Correspondence:** Esra Yilmaz

The vector-borne zoonotic parasite *Dirofilaria repens* causes cutaneous dirofilariosis. In humans, it can manifest as skin nodules in several anatomical regions or subconjunctival infections. Present in the Mediterranean area, many parts of Asia and presumably also in Africa, *D. repens* is apparently expanding its distribution to previously non-endemic areas in the old world. In Hongkong, a new species, *Candidatus* Dirofilaria hongkongensis, has been reported to cause cutaneous and subconjunctival infections of humans. The objectives of this study were to compare mitochondrial genomes from these parasites and to obtain data suitable for population genetic studies. Complete mitochondrial genomes of four adult worms from Italy, Croatia and India were obtained by either PCR followed by Sanger sequencing or Illumina MiSeq. According to cytochrome oxidase I sequences, worms from Europe and India were identified as *D. repens* and *C*. D. hongkongensis, respectively. This is the first report of *C*. D. hongkongenis from the Indian subcontinent. The mitochondrial genomes of *D. repens* and *C*. D. hongkongensis are essentially organized like those of other spirurida encoding 2 rRNAs and 12 proteins but lacking the *atp8* gene present in most animal mitochondrial genomes. An approximately 2.5 kb fragment was amplified from *Dirofilaria* positive canine blood samples or macrofilaria from Europe (n = 42), Thailand (n = 2) and Vietnam (n = 1) and analyzed together with the corresponding regions of the full-length genomes. In contrast to the very high similarity between the European and Vietnamese samples, samples from India (*C*. D. hongkongensis) and Thailand were only distantly related to the European *D. repens* samples. Notably, genetic differences between these three Asian samples were higher than those observed within *D. repens*. Phylogenetic analysis did not support the current taxonomy of the Onchocercidae but was in agreement with other recent molecular studies using multi-locus analysis. *D. repens* and *C.* D. hongkongensis sequences clustered together and were most closely related to *Dirofilaria immitis*. In conclusion, differences between *Dirofilaria* spp. were considerably high while *D. repens* was shown to be genetically quite homogenous. Analysis of mitochondrial sequences supports the hypothesis that *C*. D. hongkongensis represents a distinct species and suggests that samples from Thailand might represent another cryptic species or a genetically diverged *C.* D. hongkongensis population. Investigations on a larger geographic scale including representative numbers of samples from regions not analyzed so far as well as development of microsatellite markers for fine mapping would increase our understanding of the population genetics of *D. repens*.

## TOPIC 3: Dirofilarioses (Veterinary Medicine)

### A9 Use of histochemical analysis for updates about canine filarioids upon new cases in two dog shelters in the surrounding of Bucharest, Romania

#### Cristian-Ionut CN Florea^1,2^, Poliana Gh Tudor^1^, Stefan P Olaru^2^, Anca M Dobrica^2^

##### ^1^Department of Parasitology and Parasitic Diseases, Faculty of Veterinary Medicine, University of Agronomical Sciences and Veterinary Medicine, Bucharest, 050097, Romania; ^2^Praxis Vetlife, Bucharest, Romania, 021374

###### **Correspondence:** Cristian-Ionut CN Florea (florea_christian@yahoo.com)

Dogs represent the main natural reservoir for numerous helminths, including some species of filaria that have microfilariaes circulating in the blood flow. Of these, the most known canine filarioid species are *Dirofilaria immitis*, *D. repens*, *Acanthocheilonema reconditum* and *A. drancunculoides* transmited by different vectors (i.e. mosquitoes, fleas, lice and ticks) [1]. In Romania, stray dogs still remain an unsolved issue, despite the efforts made by the authorities in the attempt to gather and place them in shelters towards adoption. The aim of this study is the screening of the infestation with filarioids in new cases brought in two enroled dog shelters surrounding Bucharest, Romania. During November 2014 and October 2015, 282 stray dogs have been brought in two shelters near Bucharest and have been tested. Morphometric analysis of microfilariae canine blood from the enrolled dogs were made by Knott’s modified test. For the detection of the *D. immitis* antigens it was used an in-clinic rapid assay test based on enzyme immunoassay technique (SNAP® 4Dx®, IDEXX Laboratories, Inc., Westbrook, ME, USA). The identification of the species was made by a histochemical technique to demonstrate acid phophatase activity patterns in the microfilariae, using a comercial kit test (Leucognost SP®, Merck, Darmstadt, Germany) in accordance with the manufacturer’s instructions. Out of 282 enrolled dogs, 32.62% (n = 92) were positive for at least one filarial species. The modified Knott’s test showed the presence of circulating microfilariae in 78 samples (27.66%), and the serum of 67 samples was positive for *D. immitis* antigens using an immunoenzymatic assay. As a result of the histochemical technique there were identified three species of microfilariae and the global prevalence was 23.76% (n = 67) *D. immitis*, 9.57% (n = 27) *D. repens* and 0.71% (n = 2) *Acanthocheilonema spp*. In addition, coinfection with *D. immitis* and *D. repens* was found in four samples. Morphometric evaluation showed the following measurements of the length and width of microfilariae: 298.27/5.9 μm of *D. immitis*, 358.81/7.2 μm of *D. repens* and 264/4.6 μm of *Acanthocheilonema spp*. The results of this study highlight the presence of three species of filarioids from the dogs brought to the enrolled shelters with a high prevalence of *D. immitis* (23.75%). This raises public health concern that merits more consideration by both veterinarians and physicians.

**References**

1. Magnis J., Lorentz S., Guardone L., Grimm F., Magi M., Naucke T.J., Deplazes P. - Morphometric analyses of canine blood microfilariae isolated by the Knott’s test enables *Dirofilaria immitis* and *D. repens* species-specific and *Acanthocheilonema* (syn. *Dipetalonema*) genus-specific diagnosis. Parasit Vectors. 2013; 6:48.

### A10 Investigations on *Dirofilaria repens* infection in Polish dogs – looking for the objective features of the infection

#### Artur Dobrzyński^2^, Maciej Klockiewicz^1^, Magdalena Wysmołek^1^, Michał Czopowicz^3^, Marta Parzeniecka-Jaworska^4^, Joanna Nowakowska^5^, Ewa Długosz^1^

##### ^1^Division of Parasitology and Invasiology, Faculty of Veterinary Medicine, Warsaw University of Life Sciences - SGGW, Ciszewskiego St. 8, 02-786 Warsaw, Poland; ^2^Department of Small Animal Diseases with Clinic, Faculty of Veterinary Medicine, Warsaw University of Life Sciences - SGGW, Ciszewskiego St. 8, 02-786 Warsaw, Poland; ^3^Laboratory of Veterinary Epidemiology and Economics, Faculty of Veterinary Medicine, Warsaw University of Life Sciences, Ciszewskiego St. 8, 02-786 Warsaw, Poland; ^4^Department of Pathology and Veterinary Diagnostics, Faculty of Veterinary Medicine, Warsaw University of Life Sciences - SGGW, Ciszewskiego St. 8, 02-786 Warsaw, Poland; ^5^Bayer Animal Health, Aleje Jerozolimskie 158, Warsaw, Poland

###### **Correspondence:** Maciej Klockiewicz (maciej_klockiewicz@sggw.pl)

The skin dirofilariosis caused by *D. repens* has been recognized as an emerging disease in Polish dogs. Since first cases were diagnosed almost 10 years ago, now the infection is considered as an increasing epidemiological problem. The extensity of infection in some areas in Poland was estimated over 12-36% [1,2] within the local dogs’ populations. Veterinarians have found this infection as a real threat, so general aim of this research was to find objective features of the infection which could be used to establish the treatment algorithm for vets. The investigation was based on cases reported to Small Animal Clinic of the Warsaw Faculty of Veterinary Medicine as well as of those admitted to other veterinary clinics of Warsaw area. There were 428 dogs preselected (suspected for dirofilariosis) included to this research. Animals underwent physical examinations and blood tests (morphology and biochemistry). At the end of the study results of this preselected group were compared with results obtained from finally diagnosed – infected dogs. Microfilariae were found in 42.8% of examined dogs. Subsequently, PCR and ELISA tests were performed to confirm the infection in possibly infected ones. PCR with differential primers was performed to reveal parasite DNA in blood [3]. ELISA tests were based on adult *D. repens* somatic antigens to detect specific IgG in infected dogs. PCR revealed the additional 8.8% infected dogs. PCR tests also confirmed that all individuals were infected with *D. repens*. Results of ELISA indicate that *D. repens* infection results in high specific IgG titers in more than 80% of infected dogs. ELISA allowed to diagnose over 1/3 additional infected individuals, which have been previously found negative (by blood smear). The blood morphology and biochemistry revealed statistically significant erythropenia, limphopenia, thrombocytopenia, reduced haematocrit, and increased levels of alkaline phosphatase and creatinine in infected dogs. Results suggest that infection is associated with general symptoms and problems of liver and kidneys.

Additionally, the comparison between infected and not-infected groups showed that skin dirofilariosis was more often (2.6x) found in dogs which did not received any anti ecto-parasite treatment. The results were used to set up the treatment algorithm for practitioners who are not familiar with this newly emerged disease. It is allowed to suspect infection when similar blood results are obtained, and afterwards PCR and ELISA should be performed to check false-negative patients. It is concluded that such algorithm may help in prevention of this zoonotic infection in Poland.

**References**

1. Osińska B, Demiaszkiewicz AW, Pyziel AM, Kuligowska I, Lachowicz J, Dolka I. Prevalence of Dirofilaria repens in dogs in central-eastern Poland and histopathological changes caused by this infection. Bull Vet Inst Puławy. 2014; 58: 35-39. DOI: 10.2478/bvip-2014-0006

2. Demiaszkiewicz AW, Polańczyk G, Osińska B, Pyziel AM, Kuligowska I, Lachowicz J, Sikorski A. The prevalence and distribution of Dirofilaria repens in dogs in the Mazovian Province of Central-Eastern Poland. Ann Agric Environ Med. 2014; 21: 703–706.

3. Gioia G, Lecová L, Genchi M, Ferri E, Genchi C, Mortarino M. Highly sensitive multiplex PCR for simultaneous detection and discrimination of Dirofilaria immitis and Dirofilaria repens in canine peripheral blood. Vet Parasitol. 2010; 172: 160–163.

### A11 Awareness and strategies about canine heartworm (*Dirofilaria immitis*) infection in private practices in Greece: preliminary results of an ongoing questionnaire survey

#### Anastasia Diakou^1^, Mathios Mylonakis^2^, Zoe Polizopoulou^3^, Christos Koutinas^2^

##### ^1^Laboratory of Parasitology and Parasitic Diseases, School of Veterinary Medicine, Faculty of Health Sciences, Aristotle University of Thessaloniki, Thessaloniki 54124, Greece; ^2^Companion Animal Clinic, School of Veterinary Medicine, Faculty of Health Sciences, Aristotle University of Thessaloniki, Thessaloniki 546 27, Greece; ^3^Diagnostic Laboratory, School of Veterinary Medicine, Faculty of Health Sciences, Aristotle University of Thessaloniki, Thessaloniki 546 27, Greece

###### **Correspondence:** Anastasia Diakou (diakou@vet.auth.gr)

Heartworm (HW) infection of dogs is highly prevalent in some areas of Greece [1], but information about the prevention and treatment strategies implemented in the clinical setting is limited. In order to evaluate the perception of veterinarians on the prevalence and their experience on diagnosis, treatment and prevention of HW, a questionnaire survey was designed. The questionnaire was distributed by e-mail to the veterinary practitioners registered to the two major Hellenic veterinary scientific societies. Twenty questions were included, investigating the frequency of HW in each practice and the routinely implemented strategies on diagnosis, treatment and prevention. Until today, 134 questionnaires have been completed; 51.5% from the Northern and Central Greece (NCG) and 48.5% from the rest of the country (RC, continental and insular). The percentage of veterinarians reporting that they see at least one HW case per month, trimester, semester and year was 22.3%, 18.5%, 16.1% and 16.9%, respectively, while no cases of HW was reported from 26.1% of the participants. The criteria for suggesting prevention included the geographical area where the animal lived (88.8%), its lifestyle (72%), breed (13%), and the owner’s compliance (41.1%). Most of the veterinarians (61%) suggest prevention measures all year round, while some (35.5%) only during the warm season of the year. Regarding treatment, 50% of the veterinarians consider as first choice the protocol endorsed by the American Heartworm Society (AHS) and the *European Scientific* Counsel *Companion Animal Parasites (ESCCAP)*, while 16% apply a “slow kill” protocol. For the prevention of pulmonary thromboembolism 84% of the veterinarians suggest strict activity restriction, 67% administer prednisolone while 28% use aspirin.

There is a recorded difference of awareness of HW between the NCG and RC veterinarians that could be attributed to the indication of higher prevalence of HW in NCG. Indeed, only 10.3% of the NCG veterinarians report absence of HW in their area, while the corresponding percentage in RC is 40.9%. In NCG, 73.5% of the veterinarians suggest appropriate preventive measures (endorsed by the AHS and the ESCCAP) while in RC only 42.4% suggest such measures. Moreover, 85.3% of *NCG veterinarians advise HW prevention for all dogs, while the respective percentage in RC is only 12.1%.* These results suggest that although the majority of small animal practitioners in Greece appear to generally comply with the updated guidelines on the prevention and treatment of HW, the geographical area-based perceived risk for HW substantially affects the preventive strategy implemented.

**References**

1. Diakou A, Kapantaidakis E. Epidemiology of dirofilariosis in dogs in Greece: previous and latest information. 4rth European *Dirofilaria* and *Angiostrongylus* Days (FEDAD) 2-4 July, Budapest, 2014;44.

### A12 Atypical case of subcutaneous filariosis in a cat: do we expect *Dirofilaria immitis* there?

#### Simone Manzocchi^1^, Stefano Di Palma^2^, Martina Peloso^3^, Nikola Pantchev^4^

##### ^1^Novara Day Lab, IDEXX Laboratories, SP 9, Granozzo con Monticello (NO), 28060, Italy; ^2^Animal Health Trust, Lanwades Park, Kentford, Newmarket, Suffolk, CB8 7UU, United Kingdom; ^3^Ambulatorio Veterinario, Via Terraglio 194, Preganziol 31022, Treviso, Italy; ^4^IDEXX Laboratories, Mörikestr. 28/3, D-71636 Ludwigsburg, Germany

###### **Correspondence:** Simone Manzocchi (simone.mazocchi@gmail.com)

Subcutaneous dirofilariosis is a well-known disease caused mainly by *Dirofilaria repens* and described in several mammalian species including human, dog and cat [1]. Additionally, early developing stages of the heartworm, *Dirofilaria immitis,* are rarely reported in subcutaneous localization from humans and dogs. To our knowledge, evidence of this condition has not been described in the cat yet, even if the feline host can be affected either by the classic adult-related heartworm form or heartworm-associated respiratory disease (HARD) caused by immature stages. A 2 year- old, spayed male cat was presented for three subcutaneous nodules on the head and trunk. The cat lived in Northern Italy and was regularly vaccinated and treated monthly with an antiparasitic spot on formulation containing selamectin (Stronghold®, Pfizer). One of the three nodules was surguically excided and examined. Histology showed in the subcutis the presence of a nodular lesion characterized by a severe inflammatory infiltrated composed by macrophages, small lymphocytes, with fewer eosinophils and mast cells, supported by a proliferation of mature fibroblasts (fibrosis). Inflammatory cells were multifocally surrounding sections of parasites identified as nematodes. The parasites were characterized by a thick cuticle with a smooth external surface, prominent and large lateral chords and a polymyarian-coelomyarian musculature. Microscopic features were compatible with *D. immitis* morphology. [2] After extraction from the paraffin block, DNA of the parasite was amplified with a PCR (ribosomal 5.8S-ITS2-28S region), the PCR product were purified, cloned and thereafter sequenced. A BLAST search revealed 97% identitiy to *D. immitis* isolate EU182331 and only 79% of identity the next related sequence of *Dirofilaria* genus (*D. repens*). The cat tested negative for *D. immitis* antigenemia and the two remaining nodules disappeared spontaneously in a few months. Identification of a filaroid nematode with smooth cuticles in the subcutaneous tissues can be challenging. All species of the genus *Dirofilaria* are characterized by cuticular ridges, except from *D. immitis* and *D. lutrae* [2], with the latter described so far only in USA in the North American river otter. The parasite in the present case most likely represents an immature stage of *D. immitis* which developed in the subcutis (L3-L4) and was successively entrapped in this localization. The immunity of the cat, which is not a suitable definitive host for *D. immits*, likely played a role in preventing migration of the immature stage to the pulmonary arteries.

To author’s knowledge this is the first reported case of subcutaneous localization of *D. immits* in a feline host.

**References**

1. Manzocchi S, Lendner M, Piseddu E, Morabito S, Sebastiani V, Daugshies A, Pantchev N. Nodular presentation of dirofilaria repens infection in a cat mimicking a fibrosarcoma. Vet Clin Pathol., 2015, In press.

2. Orihel CT and Eberhard ML. Zoonotic Filariasis. Clin Microbiol Rev., 1998, 11: 366–381.

### A13 In field retrospective study of „slow-kill “treatment efficiency on heartworm positive dogs in general practises in Serbia

#### Nenad Milojković^1^, Momčilo Aranđelović^2^, Ljubomir Ćurčin^3^

##### ^1^Veterinary Clinic “Vet Centar“, 11000 Belgrade, Serbia; ^2^Veterinary Clinic “Vet Alfa“, 11000 Belgrade, Serbia; ^3^Veterinary Clinic “Intervet“, 11000 Belgrade, Serbia

###### **Correspondence:** Nenad Milojković (nenad.milojkovic66@gmail.com)

Northern parts of Serbia are hyperendemic for *Dirofilaria immitis*. A lot of suburban and rural areas may have overall prevalences up to 50% in dogs, and treating them is of great concern for veterinarians in the field. Two limitations make adulticide treatment almost impossible: 1. Immiticide® is not available on the Serbian market and ordering it abroad is expensive for the majority of owners whose dogs are confirmed as Heartworm (HW) positive; 2. It is very difficult, especially in the countryside to do appropriate diagnostics (echocardiography) in order to estimate prognosis after melorsamine treatment. Therefore, the „slow kill“ treatment is the only reliably choice for the majority of veterinarians in the field. We have gathered data about HW positive dogs from eleven general practises. The total number of antigen positive dogs was 258. Owners of only 105 dogs were interested to treat their pets. Seven of them have stoped visiting their veterinarians after one month, and 32 dogs with severe disease (respiratory distress, right sided heart failure, caval sindrome) died within the first 3 months after diagnostistics. A total number of 66 dogs, with mild and moderate disease, have continued with „slow- kill“ treatment. Schedule for treatment was intermittent application of doxicyclin (10 mg/kg every third month) and prophylactic dose of ivermectin (10 mcg/kg every 15 days). Therapy was given until the first negative antigen test. During the first 3 months 9 (13.64%) patients became antigen negative, between 3 and 6 months 3 (4.54%), between 6 and 9 months 29 (43.94%) and 14 (21.24%) between 9 and 12 months; 11 (16.64%) dogs become negative after more than 12 months. All dogs from the last group did not visit veterinarians regularly and were not on continuous therapy. We also want to remark that a lot of dogs in the study were not tested each month. Most of them were retested twice annualy, due to financial capabilities of their owners. Data from the study shows frequent owners neglecting to do any further dignostics and treatment of their HW positive dogs in suburban and rural areas. Those dogs persist as a reservoir for the disease. This fact demands more active education of the owners. On the other hand, dogs of committed owners, with mild and moderate disease, which are going to „slow kill“ treatment, have a good chance to be cured.

**References**

1. Grandi G, Quintavalla C, Mavropoulou A, Genchi M, Gnudi G, Bertoni G, Kramer L. A combination of doxycycline and ivermectin is adulticidal in dogs with naturally acquired heartworm disease (*Dirofilaria immitis*)*Veterinary Parasitology, Volume 169, Issues 3–4, 11 May 2010, Pages 347-351*

2. Mavropoulou A, Gnudi G, Grandi G, Volta A, Kramer L, Quintavalla C. Clinical assessment of post-adulticide complications in *Dirofilaria immitis*-naturally infected dogs treated with doxycycline and ivermectin**;** *Veterinary Parasitology, Volume 205, Issues 1–2, 15 September 2014, Pages 211-215*

3. Venco L, McCall J, Guerrero J, Genchi C. Efficacy of long-term monthly administration of ivermectin on the progress of naturally acquired heartworm infections in dogs, *Veterinary Parasitology, Volume 124, Issues 3–4, 5 October 2004, Pages 259-268*

4. McCall J, Genchi C, Kramer L, Guerrero J,. Dzimianski M, Supakorndej P, Mansour A, McCall S, Supakorndej N, Grandi G, Carson B. Heartworm and Wolbachia: Therapeutic implications. Veterinary Parasitology, 2008, 158, 204–214.

5. http://www.esccap.org Control of vector- borne diseases in dogs and cats; ESCCAP Guideline 05 second edition – October 2012.

6. http://www.heartwormsociety.org Current Canine Guidelines.

7. Rawlings C., Calvert C., Heartworm disease, in Ettinger S., Elsevier Sounders, 1995, (1046-1067)

8. Atkins C., Canine heartworm disease; Feline heartworm disease, in Ettinger S., Elsevier Sounders, 2005, (1118 – 1144).

## TOPIC 4: Dirofilarioses (Veterinary Medicine/Epidemiology/Tropical Parasitology)

### A14 The true story of *Dirofilaria* in the Czech Republic

#### Barbora Mitková^1,2^, Marcela Novotná^3^, Jana Juránková^1^, Lada Hofmannová^1^, Dwight D. Bowman^3^, David Modrý^1,2,4^

##### ^1^Department of Pathology and Parasitology, University of Veterinary and Pharmaceutical Sciences, Brno 61242, Czech Republic; ^2^CEITEC VFU, University of Veterinary and Pharmaceutical Sciences, Brno 61242, Czech Republic; ^3^Department of Microbiology & Immunology, College of Veterinary Medicine Cornell University, Ithaca, NY 14853, USA; ^4^Biology Centre, Institute of Parasitology, Czech Academy of Sciences, České Budějovice 370 05, Czech Republic

###### **Correspondence:** Barbora Mitková (bmitkova@pobox.sk)

The first autochthonous infection of *Dirofilaria repens* and *Dirofilaria immitis* in the Czech Republic were reported in 2006 [1] using several diagnostic methods for detections of these parasites. Since then, *Dirofilaria* infection was repeatedly reported in dogs [2] and, recently, *D. repens* was detected also in mosquitoes [3] and in humans [4]. The presence of *D. immitis* in the Czech Republic was established only on detection of antigen using the commercially available test. In past 10 years, detection of *D. immitis* microfilariae, PCR detection or clinical case of canine dirofilariosis caused by *D. immitis* were not reported from the Czech Republic. The aim of presented survey was to confirm or exclude the autochthonous infection of *D. immitis* in dogs from the Czech Republic and to determine the extent of endemic distribution of *D. repens* within the Czech Republic. A total number of 392 blood samples from dogs were examined using the modified Knott test, IDEXX SNAP® 4Dx® test and PCR amplifying the fragment of COI gene of filarial nematodes [5]. Only *D. repens* was detected by Knott test and /or by PCR with prevalence 6.4% (25/392). Six out of 25 positively diagnosed dogs had no travel history outside the Czech Republic, so the autochthonous infection was proven in 3.4% animals. Almost all positive dogs had originated from Southern Moravia region except a single one, which was from Zlín region, 100 km north of other positive localities. *D. repens* prevalence demonstrated in our sample set is lower than previously published (9–24%), however, distribution of positive animals corresponds well with published presence of *D. repens* positive mosquitoes and with occurrence of cases of autochthonous human dirofilariosis from the same region. Our study confirmed the endemic occurrence of *D. repens* in the region of Southern Moravia in the Czech Republic. Importantly, no *D. immitis* was detected. Based on these results, and considering total absence of published clinical cases, microfilariae or PCR detection of *D. immitis* in the Czech Republic, we strongly recommend not to consider the Czech Republic as currently endemic for this parasite.

This study was supported by COST CZ LD14048; survey was organized in the framework of the EurNegVec COST Action TD1303.

**References**

1. Svobodová Z, Svobodová V, Genchi C, Forejtek P. The first report of authochthonous dirofilariosis in dogs in the Czech Republic. Helminthologia. 2006; 43:242–245. doi:10.2478/s11687-006-0046-5

2. Dobešová R, Svobodová V. Progressive spread of *Dirofilaria* infections in dogs along rivers in the southeastern Czech Republic. In Morchón R., Simón F., Montoya J. A., Genchi C. 2nd European Dirofilaria Days, Programe and abstract SEDD 2009; O 4, p.190.

3. Rudolf I, Šebesta O, Mendel J, Betášová L, Bocková E, Jedličková P, Venclíková K, Blažejová H, Šikutová S, Hubálek Z. Zoonotic *Dirofilaria repens* (Nematoda: Filarioidea) in *Aedes vexans* mosquitoes, Czech Republic. Parasitol Res. 2014; 113:4663–4667. doi:10.1007/s00436-014-4191-3

4. Matějů J, Chanová M, Modrý D, Mitková B, Hrazdilová K, Žampachová V, Kolářová L. *Dirofilaria repens*: emergence of autochthonous human infections in the Czech Republic (case reports). BMC Infect Dis. 2016; 16:171. doi:10.1186/s12879-016-1505-3

5. Casiraghi M, Anderson TJ, Bandi C, Bazzocchi C, Genchi C. A phylogenetic analysis of filarial nematodes: comparison with the phylogeny of *Wolbachia* endosymbionts. Parasitology. 2001; 122:93–103. doi:10.1017/S0031182000007149

### A15 Fighting endemicity of *Dirofilaria* spp. in Austria

#### Michael Leschnik

##### Department for Companion Animals and Horses Veterinary University Vienna, Vienna 1210, Austria

###### **Correspondence:** Michael Leschnik (michael.leschnik@vetmeduni.ac.at)

**Fig. 1 (abstract A15).**
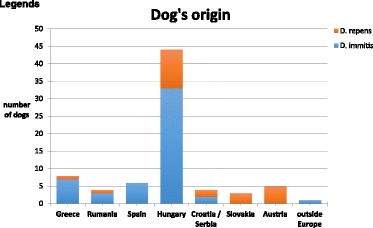
Origin of imported cases of canine dirofilariosis to Austria

**Fig. 2 (abstract A15).**
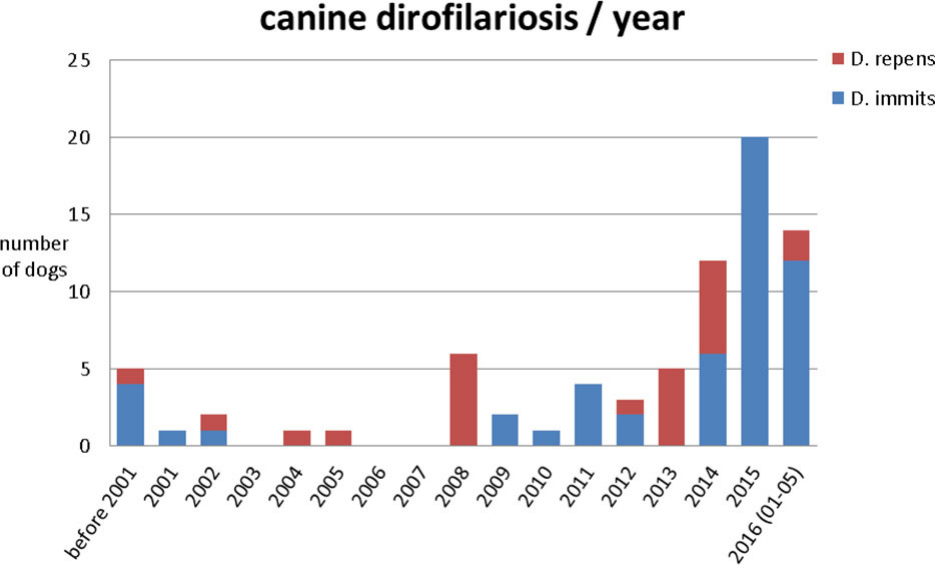
Number of diagnosed cases of canine dirofilariosis per year in Austria

Canine dirofilariosis has rarely been diagnosed in Austria before 2008. All dogs had a history either originating from an endemic country or staying abroad for a certain time. Dogs were identified by accidental finding of microfilaria in blood or urine samples or by the directed detection of adult *D. immitis* or *D. repens* [1, 2]. From 2008 on case numbers increased rapidly regarding both infections. The typical origin from Mediterranean countries in dogs with heartworm disease has been replaced by the origin from Eastern countries, led by far by cases from Hungary (Fig. 1). Several animal welfare associations located in Austria financially support foreign animal shelters in neighbouring countries and organize dog importation to Austria and Germany on a large scale. Unfortunately, most of these animals are not tested for dirofilariosis prior to importation and they are not protected by microfilarizides to avoid local transmission to mosquitos. First canine cases of *D. repens* infections with probable autochthonous background have been diagnosed in Austria in 2008 [3]. In 2014 first detection of *D. repens* in vector mosquitos has been reported [4] and confirmed by additional autochthonous canine cases. An obvious increase of imported dogs from eastern countries to Austria has been recognized within the last five years, concurrently canine heartworm disease cases increased markedly, too (Fig. 2). Several criteria turned out to be important in the consultation talk to the dog’s owner for the decision to have these animals on therapy:

1. Several animal welfare association members refuse heartworm therapy in dogs due to possible side effects. Side effects and lethality rates were massively overstated and erroneously reported to the owners.
2. Estimated costs are high, especially when following the guidelines from the American Heartworm Society
3. None of the owners was informed about the possible influence of importing infected dogs to Austria regarding endemicity and zoonotic hazards.

To offer a safe and affordable therapeutic regime, a modified scheme has been introduced to these animals including two injections of melarsomine three days apart, and oral medication of macrocyclic lactones and doxycyclin. Transmission risk was reduced immediately and 22/26 became negative in the antigen test within 4-8 months after melarsomine injections. Informing and advising animal welfare associations and dog owners as well as identification of imported and infected animals and the rapid onset of a safe therapy is the only way to delay or even avoid heartworm disease becoming endemic in Austria.

**References**

1. Leschnik M, Löwenstein M, Edelhofer R, Kirtz G. Imported non-endemic, arthropod-borne and parasitic infectious diseases in Austrian dogs. Wien Klin Wochenschr. 2008;120 (19-20 Suppl 4):59–62.

2. Fuehrer HP, Auer H, Leschnik M, Silbermayr K, Duscher G, Joachim A. Dirofilaria in Humans, Dogs, and Vectors in Austria (1978-2014)-From Imported Pathogens to the Endemicity of Dirofilaria repens. PLoS Negl Trop Dis. 2016; 10(5):e0004547.

3. Duscher G, Feiler A, Wille-Piazzai W, Bakonyi T, Leschnik M, Miterpáková M, Kolodziejek J, Nowotny N, Joachim A. Detection of Dirofilaria in Austrian dogs. Berl Munch Tierarztl Wochenschr. 2009; 122(5-6):199-203.

4. Silbermayr K, Eigner B, Joachim A, Duscher GG, Seidel B, Allerberger F, Indra A, Hufnagl P, Fuehrer HP. Autochthonous Dirofilaria repens in Austria. Parasit Vectors. 2014; 7:226.

### A16 Detection of *Dirofilaria immitis* antigen in red foxes (*Vulpes vulpes*) from Portugal

#### Ana Margarida Alho^1^, Helder CE Cortes^2^, Ana Patrícia Lopes^3,4^, Maria João Vila-Viçosa^2^, Luís Cardoso^3^, Silvana Belo^5^, Luís Madeira de Carvalho^1^

##### ^1^CIISA, Faculty of Veterinary Medicine, ULisboa, Lisboa, Portugal; ^2^Victor Caeiro Laboratory of Parasitology, Instituto de Ciências Agrárias e Ambientais Mediterrânicas (ICAAM), University of Évora, Évora, Portugal; ^3^Department of Veterinary Sciences, School of Agrarian and Veterinary Sciences, University of Trás-os-Montes e Alto Douro (UTAD), Vila Real, Portugal; ^4^Animal and Veterinary Research Centre (CECAV), UTAD, Vila Real, Portugal; ^5^Medical Parasitology Unit, Global Health and Tropical Medicine, Instituto de Higiene e Medicina Tropical, Universidade Nova de Lisboa, Lisboa, Portugal

###### **Correspondence:** Luís Madeira de Carvalho (madeiradecarvalho@fmv.ulisboa.pt)

Cardiopulmonary dirofilariosis caused by *Dirofilaria immitis*, is a zoonotic mosquito-borne disease with a worldwide distribution and a potentially severe outcome in companion animals. Wild canids constitute a reservoir for many vector-borne diseases, by harbouring their vector-transmitted etiological agents [1, 2]. Red foxes (*Vulpes vulpes*) are the most abundant wild carnivores in Europe, their populations are increasing and they are invading many urban areas due to a good adaptation to human environments [1]. Portugal is an endemic country for dirofilariosis in domestic dogs [3], although information on the occurrence of *D. immitis* in wild canids is sparse. An epidemiological survey was conducted to investigate the prevalence of *D. immitis* in red foxes from Portugal and to evaluate their potential role in the epidemiology of dirofilariosis. Blood (n = 94) or meat juice (n = 25) were obtained from 119 wild red fox carcasses shot during the official hunting season or killed on the road due to traffic accidents between 2008 and 2010. These animals came from eight districts of northern (Braga, Bragança, Porto, Viana do Castelo and Vila Real), central (Aveiro) and southern Portugal (Évora and Setúbal). *D. immitis* circulating antigens were detected using a commercially available enzyme-linked immunosorbent assay (ELISA) antigen kit, (WITNESS® Dirofilaria; Synbiotics, Europe). Out of the 119 foxes, 10 (8.4%; CI: 4.1-14.9%) were found infected with *D. immitis*, with positive animals found in five districts (Braga, Bragança, Évora, Viana do Castelo and Vila Real), in northern and southern areas of Portugal. One of the samples that were positive to *D. immitis* was obtained with meat juice, a finding which suggests that it could be used as an alternative sample to serum for the antigen detection of antigen, in post-mortem analysis. The present report demonstrates that infection with *D. immitis* is prevalent in red fox populations in Portugal, showing an increase of prevalence compared with recent reports [4, 5] and suggesting a role of these animals as potential reservoir hosts for domestic pets and even to humans. Given the complex interaction between wildlife and domestic animals, humans and parasites, a robust health risk surveillance assessment should be implemented in Portuguese fox population to allow a better management of its vector-borne infections and diseases, in line with the 'One Health' concept.

**Funding**

PhD Research Grant SFRH/BD/85427/2012; Projects UID/CVT/00276/2013 CIISA-FMV-ULisboa and GHTM-UID/Multi/04413/2013, supported by Fundação para a Ciência e a Tecnologia (FCT), Portugal.

**References**

1. Otranto D, Cantacessi C, Pfeffer M, Dantas-Torres F, Brianti E, Deplazes P, Genchi C, Guberti V, Capelli G. The role of wild canids and felids in spreading parasites to dogs and cats in Europe. Part I: Protozoa and tick-borne agents. Vet Parasitol. 2015; 213:12–23.

2. Otranto D, Cantacessi C, Dantas-Torres F, Brianti E, Pfeffer M, Genchi C, Guberti V, Capelli G, Deplazes P. The role of wild canids and felids in spreading parasites to dogs and cats in Europe. Part II: Helminths and arthropods. Vet Parasitol. 2015; 213:24–37.

3. Alho AM, Landum M, Ferreira C, Meireles J, Gonçalves L, de Carvalho LM, Belo S. Prevalence and seasonal variations of canine dirofilariosis in Portugal. Vet Parasitol. 2014; 206:99–105.

4. Eira, C, Vingada, J, Torres, J, Miquel, J. The Helminth community of the red fox, *Vulpes vulpes*, in Dunas de Mira (Portugal) and its effect on host condition. Wildl Biol Pract. 2006, 2(1): 26–36.

5. Ferreira I Rastreio Sorológico de alguns agentes de Zoonoses em Canídeos Silvestres no Norte de Portugal. 2010, MSc Dissertation, FMV-TUL, 132 pp.

### A17 In search of *Dirofilaria immitis* in domestic dogs from Luanda

#### Ana Margarida Alho^1^, Hugo Vilhena^2,3,4^, Ana Cristina Oliveira^5^, Sara Granada^5^, Ana Patrícia Lopes^4,6^, Silvana Belo^7^, Luís Madeira de Carvalho^1^, Luís Cardoso^6^

##### ^1^CIISA, Faculty of Veterinary Medicine, ULisboa, Lisboa, Portugal; ^2^Animal and Veterinary Research Centre (CECAV), University of Trás-os-Montes e Alto Douro (UTAD), Vila Real, Portugal; ^3^Department of Veterinary Medicine, Escola Universitária Vasco da Gama, Coimbra, Portugal; ^4^Hospital Veterinário do Baixo Vouga, Águeda, Portugal; ^5^Clínica Casa dos Animais, Luanda, Angola; ^6^Department of Veterinary Sciences, School of Agrarian and Veterinary Sciences, UTAD, Vila Real, Portugal; ^7^Medical Parasitology Unit, Global Health and Tropical Medicine, Instituto de Higiene e Medicina Tropical, Universidade Nova de Lisboa, Lisboa, Portugal

###### **Correspondence:** Ana Margarida Alho (madeiradecarvalho@fmv.ulisboa.pt)

Angola is a country located in Southern Africa, with a mild semi-arid climate. Several tick-borne pathogens were recently reported in domestic dogs in the country [1, 2]. However, data on cardiopulmonary dirofilariosis, a zoonotic mosquito-borne disease that is potentially lethal to companion animals, is non-existent. To assess the potential occurrence of infection with *Dirofilaria immitis* in canids, 103 domestic dogs presented to a veterinary medical centre in Luanda were evaluated. Luanda was chosen as it is both the capital and the largest city in the country. Based on physical examination and clinicopathological data, 50 dogs were classified as apparently healthy and 53 as clinically suspected of a canine vector-borne disease. The population tested comprised 62 males and 41 females, with ages ranging from 3 months to 14 years (median: 1.0 year; interquartile range: 0.6-4.0). A commercially available *D. immitis* antigen test (WITNESS® Dirofilaria; Synbiotics, Europe) was used. Plasma was heat treated (at 103 °C for 10 min in a dry heat block and the resultant clot was centrifuged) to destroy potential blocking antibodies or inhibitors, favouring accurate diagnosis [3]. Out of the 103 dogs tested, none was positive in the antigen test. Commercially available *D. immitis* antigen kits are highly specific and sensitive diagnostic methods, recommended for mass population screening [4]. Despite the various pathogens previously described in this population [1, 2], *D. immitis* antigen were not found in any of these dogs. *D. immitis* has already been reported in other sub-Saharan African countries, including Kenya [5], Mozambique [6] and Zambia [7], although to the best of the authors’ knowledge there is no report of *D. immitis* in Angola. Nonetheless, we cannot exclude the existence of *D. immitis* or another related species (*Dirofilaria repens*, for example) that is not detectable with this routine test. Unfortunately, no blood was available to perform the modified Knott’s technique to assess potential microfilaremia. Considering the zoonotic risk of this parasite and the presence of potential vectors, further studies are needed to characterize the current epidemiological scenario of filarial species in vertebrate hosts and vector insects in Angola.

**Funding**

PhD Research Grant SFRH/BD/85427/2012; Projects UID/CVT/00276/2013 CIISA-FMV-ULisboa and GHTM-UID/Multi/04413/2013, supported by Fundação para a Ciência e a Tecnologia (FCT), Portugal.

**References**

1. Vilhena H, Granada S, Oliveira AC, Schallig HD, Nachum-Biala Y, Cardoso L, Baneth G. Serological and molecular survey of *Leishmania infection* in dogs from Luanda, Angola. Parasit Vectors., 2014, 7: 114.

2. Cardoso L, Oliveira AC, Granada S, Nachum-Biala Y, Gilad M, Lopes AP, Sousa SR, Vilhena H, Baneth G. Molecular investigation of tick-borne pathogens in dogs from Luanda, Angola. Parasit Vectors., 2016, 9: 252.

3. Velasquez L, Blagburn BL, Duncan-Decoq R, Johnson EM, Allen KE, Meinkoth J, Gruntmeir J, Little SE. Increased prevalence of *Dirofilaria immitis* antigen in canine samples after heat treatment. Vet Parasitol., 2014, 206: 67–70.

4. Nelson CT, McCall JW, Carithers D. Current canine guidelines for the diagnosis, prevention, and management of heartworm (*Dirofilaria immitis*) infection in dogs. American Heartworm Society, 2014, https://www.heartwormsociety.org/

5. Bwangamoi O, Frank H. The incidence and pathology of *Dirofilaria immitis* infection in dogs in Nairobi. J Small Anim Pract. 1970;11(5):293–300.

6. Schwan EV1, Durand DT. Canine filariosis caused by *Dirofilaria immitis* in Mozambique: a small survey based on the identification of microfilariae. J S Afr Vet Assoc., 2002, 73: 124–126.

7. Siwila J, Mwase ET, Nejsum P, Simonsen PE. Filarial infections in domestic dogs in Lusaka, Zambia. Vet Parasitol., 2015; 210: 250–254.

## POSTER SESSION

### A18 A skin nodule due to *Dirofilaria repens* in a Tosa dog in Ile de France

#### Radu Blaga^1^, Virginie Daniel-Lesnard^2^, Bruno Polack^1^, Stéphanie Beurlet^3^, Coralie Martin^4^, Jacques Guillot^1^

##### ^1^Parasitology department, BioPôle d’Alfort, Ecole nationale vétérinaire d’Alfort, UPE, Maisons-Alfort, France; ^2^Clinique vétérinaire du Chêne Rouge, Chanteloup-en-Brie, France; ^3^Vebio laboratory, Arcueil, France; ^4^Muséum national d’Histoire naturelle, Paris, France

###### **Correspondence:** Jacques Guillot (jacques.guillot@vet-alfort.fr)

*Dirofilaria repens* is not a life-threatening parasite, however is one of the major differential diagnosis that must be done when blood microfilariae are detected in a dog. This was the case in a 20 month-old Tosa dog that came to the surgery consultation of the Small Animals Veterinary Hospital of Alfort, France in February 2016. The dog had a subcutaneous skin nodule on the head. The medical imaging examination, performed the day of the consultation, showed a well-defined nodule of 1.5 cm of large and 5 mm of depth, with several hyperechoic lines inside. The nodule was punctured with a small needle and polynuclear granulocytes as well as microfilariae were detected, after staining. Blood analysis revealed the presence of microfilariae both in the smear and in the sediment following the Knott technique (mean 176 larvae/mL). After the surgical removal of the skin nodule, one nematode of 8 cm of length was found inside, identified by means of PCR (ITS) as *Dirofilaria repens*. Blood analysis performed 6 weeks later, in the absence of any treatment, demonstrated a decrease of microfilariemia of 22%. The dog was imported from South of Romania, at the age of 3 months, and since then, the dog never left Ile de France region. Since both in Romania and in Ile de France region, several cases of subcutaneous dirofilariosis have been described so far in dogs and cats, it is not possible to identify with certainty the place where the initial contamination occurred. However the region of south Romania, where the dog was born is highly endemic for mosquitoes.

### A19 Serological survey of *Dirofilaria* in humans from Romania and Republic of Moldova

#### Lavinia Ciuca^1^, Rodrigo Morchón^2^, Ruxandra V. Moroti^5^, Mihaela Arbune^6^, Loredana Hurjui^7^, Roman Constantin^1^, ^1^Dumitru Acatrinei, Liviu Miron^1^, Laura Kramer^4^, Laura Rinaldi^3^, Fernando Simón^2^

##### ^1^Ion Ionescu de la Brad University of Agricultural Sciences and Veterinary Medicine Iasi, Iași, 700490, Romania; ^2^Laboratory of Parasitology, University of Salamanca, Salamanca, 37007, Spain ^3^Department of Veterinary Medicine and Animal Productions, University of Naples Federico II, Naples, 80138, Italy; ^4^Department of Veterinary Medicine, University of Parma, Parma, 43124, Italy; ^5^Carol Davila University of Medicine and Pharmacy, Bucarest, 050474, Romania; ^6^Faculty of Medicine and Pharmacy, Dunărea de Jos University of Galaţi, Galați, 800001**,** Romania; ^7^Grigore T. Popa University of Medicine and Pharmacy, Iași, 700115, Romania

###### **Correspondence:** Lavinia Ciuca (lavinia_vet1@yahoo.com)

Dirofilariosis is an emerging zoonotic infection caused by the filarial nematodes of dogs *Dirofilaria repens* and *Dirofilaria immitis* [1]. The high prevalence of both *Dirofilaria* species in dogs in Romania represent a constant threat for animal and public health. However, only few cases of human infections by *D. repens* have been reported from various regions of Romania so far. In the present study a serological screening was performed on a cohort of patients from Romania (n = 187) and Republic of Moldova (n = 263) for a total of 450 patients (166 male and 284 females; aged 7 to 78 years). Sera samples were collected from December 2015 to March 2016 and analyzed by a non-commercial IgG-ELISA for the detection of IgG anti-*D. immitis* and anti-*D. repens*. Of 187 patients from Romania 13 (6.9%) were positive for *D. immitis*, and only one patient reacted against both antigens of *D. immitis* and *D. repens*. Of 263 patients from Republic Moldova, 36 (13.6%) were positive for *D. immitis* and three (1.4%) patients recognized both antigens. Only one patient was found positive for IgG-anti *D. repens*. Moreover the results from the present study were confirmed by Western blot analysis, which gives even greater support to these results. Our results confirmed the public health significance and zoonotic impact of *Dirofilaria* infections in Romania and the Republic of Moldova. Considering that the main resevoir is represented by the microfilaremic dogs and the infections are transmitted by mosquitoes, entomological surveillance and monitoring of dogs are needed in both countries, in order to define the risk areas of infection.

**References**

1. Simón F, Siles-Lucas M, Morchón R, González-Miguel J, Mellado I, Carretón E, Montoya-Alonso JA. Human and animal dirofilariasis: the emergence of a zoonotic mosaic. Clin Microbiol Rev. 2012; 25 (3): 507–544.

### A20 Molecular investigation of possible *Dirofilaria repens* vertical transmission from queen to offspring - case report from Poland

#### Ewa Długosz^1^, Agnieszka Szmidt^2^, Artur Dobrzyński^3^, Magdalena Wysmołek^1^, Maciej Klockiewicz^1^

##### ^1^Division of Parasitology and Invasiology, Faculty of Veterinary Medicine, Warsaw University of Life Sciences – SGGW, Ciszewskiego St. 8, 02-786 Warsaw, Poland; ^2^ Out-patients veterinary clinic "Przy Forcie", Obrońców Tobruku St. 27 lok. 4, 01-494 Warsaw, Poland; ^3^ Department of Small Animal Diseases with Clinic, Faculty of Veterinary Medicine, Warsaw University of Life Sciences – SGGW, Nowoursynowska St. 159, 02-786 Warsaw, Poland

###### **Correspondence:** Ewa Długosz (ewa_dlugosz@sggw.pl)

A stray queen with her offspring was delivered to the veterinary clinic in Warsaw. The litter consisted of 3 female and 4 male kittens. The age of kittens was estimated around 8 weeks. During physical examination all of them were found in poor condition. The family was severely infested with fleas. Fecal examination results showed that the queen was infected with *Toxocara* sp., *Ancylostoma* sp., and *Dipyllidium* sp., and the offspring with roundworms and hookworms. Some of the kittens manifested diarrhea and also conjunctivitis was noticed. A blood sample was collected from the adult cat to check its general status. During the examination some individual microfilariae were found. Blood samples were then taken from three kittens and blood smears revealed the presence of single microfilariae in two of them. Regarding the severity of the circumstances the veterinarian decided to apply moxidectin/imidacloprid topical solution (Advocate®, Bayer) and other necessary treatment. Two days later the veterinarian contacted our laboratory in the Division of Parasitology at the Faculty of Veterinary Medicine. We asked for blood and serum samples which were then taken from the queen and the kittens and delivered to our laboratory. In order to confirm the infection genomic DNA was isolated from blood samples and PCR was performed [1]. PCR product specific for *D. repens* was amplified only in the sample originating from the queen which was taken before treatment. In queen and kitten blood samples which were taken after treatment PCR results were unambiguous. The presence of *D. repens* specific antibodies in all examined sera was confirmed by ELISA. The highest titer was noted in queen serum (1/25600). Titers in kitten sera were lower and ranged from 1/3200 to 1/800. Vertical transmission of filarial infections is uncommon. Only few cases of transplacental transmission of microfilariae have been reported: *Brugia phanangi* in the cat [2], *Dirofilaria immitis* in the dog [3], *Wuchereria bancrofti* and *Onchocerca volvulus* in humans [4, 5]. Our results allow to hypothesize that *D. repens* vertical transmission occurred in investigated cats. At the same time, it is very unlikely that kittens had been infected by another way at this age. In conclusion, there are many questions to be answered. What was the actual route of transmission in this particular case? What is the pattern of immune response against *D. repens* in cats? More research should be conducted in order to provide the adequate control measures to prevent skin dirofilariosis in pets and humans.

**References**

1. Gioia G, Lecová L, Genchi M, Ferri E, Genchi C, Mortarino M. Highly sensitive multiplex PCR for simultaneous detection and discrimination of *Dirofilaria immitis* and *Dirofilaria repens* in canine peripheral blood. Vet Parasitol. 2010; 172:160–136.

2. Kimmig P. Diaplacental transmission of microfilaria of the species, *Brugia pahangi*, in the cat. Z Parasitenkd. 1979; 58:181–186.

3. Mantovani A, Jackson RF. Transplacental transmission of microfilariae of *Dirofilaria immitis* in the dog. J Parasitol. 1966; 52:116.

4. Campello TR, Ferreira RS, Pires ML, De Melo PG, Albuquerque R, Araujo S, Dreyer G. A study of placentas from *Wuchereria bancrofti* microfilaraemic and amicrofilaraemic mothers. J Trop Med Hyg. 1993; 96:251–255.

5. Ufomadu GO, Sato Y, Takahashi H. Possible transplacental transmission of *Onchocerca volvulus*. Trop Geogr Med. 1990; 42:69–71.

### A21 Superficial *Dirofilaria repens* infection: a case series in Serbia

#### Aleksandar M Džamić^1^, Tanja Kalezić^2^, Ivana Čolović Čalovski^1^, Dejan Rašić^2^, Milan Cvetković^1^, Sanja Mitrović^1^

##### ^1^Laboratory of Parasitology-Mycology, Institute of Microbiology and Immunology, University of Belgrade Faculty of Medicine, 11 000 Belgrade, Serbia; ^2^Clinic for Eye Diseases, Clinical Center of Serbia, University of Belgrade Faculty of Medicine, 11 000 Belgrade, Serbia

###### **Correspondence:** Aleksandar M Džamić (dzamica@med.bg.ac.rs)

**Fig. 3 (abstract A21).**
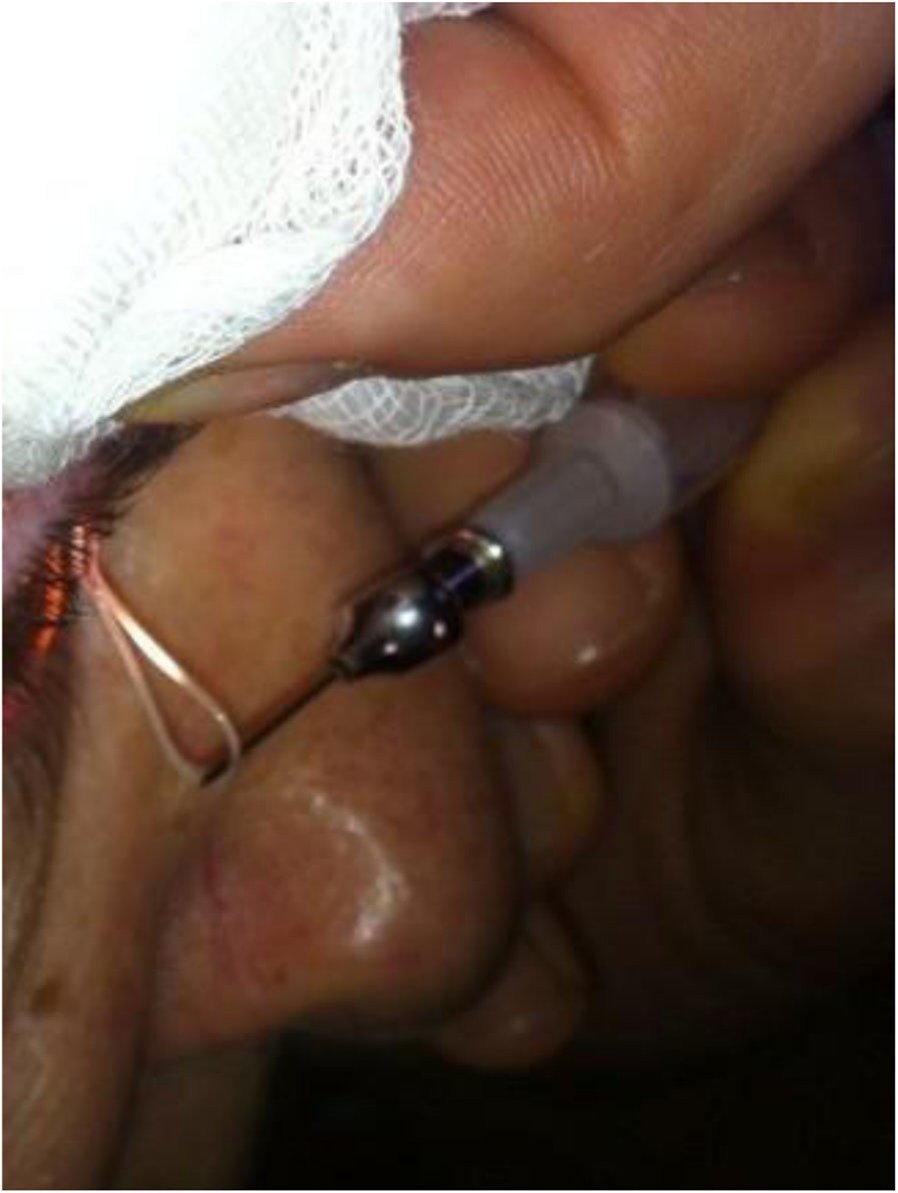
See text for description

**Fig. 4 (abstract A21).**
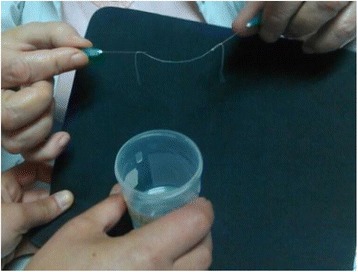
See text for description

**Fig. 5 (abstract A21).**
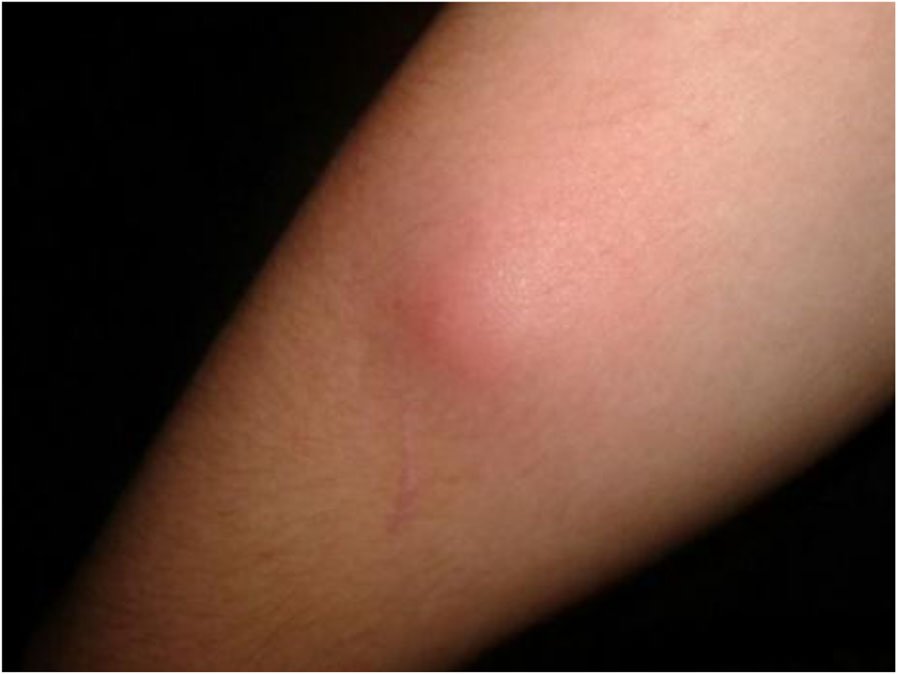
See text for description

Human dirofilariasis caused by a *Dirofilaria repens* is relatively rare zoonotic infestation, but according to the literature number of reported cases increase in Serbia, the Balkans and other European countries in the last 10 years [1, 2, 3]. Recently, the parasite was identified by molecular techniques in *Culex pipiens* and *Aedes vexans* in Serbia [4]. About 37 cases of superficial (subcutaneous and eye infections) and visceral infections were reported in Serbia till 2015 with predominant subcojnunctival and periocular infestation. Aim of this paper is to report new *D. repens* infections diagnosed in our country, and to address attention that this mostly benign infection may have serious clinical course. We present three cases of human dirofilariasis, two autochthonous and one imported, diagnosed from February 2015 till April 2016. All patients were adults, one male and two females, two with subcutaneous infection on the limbs and one with infection of the eye. The male patient, 57 years old is a resident of Belgrade who frequently travelled to Novi Pazar which is located in the southern part of Serbia. He presented with five days history of pain, swelling and redness on the anterior part of the right thigh (6 ˣ 12 cm) near inguinal area, temperature 38 ° C and eosinophilia (10.3). According to the clinical picture and ultrasound findings phlegmona and cellulites were diagnosed and ceftriaxone (2 g IV 5 days) was prescribed. Although pain and redness disappeared, and nodular swelling was clearly defined (5 cm), needle puncture was performed and white thread like 8 cm long mass was extracted and amoxicillin/clavulanic acid (100 mg twice/day for 15 days) was prescribed. Diagnosis of a *D. repens* infection was made in pathohistological preparations and the infectiologist prescribed ivermectin (200mcg/kg PO once). He has been on follow-up for two months without any signs of recurrence. At the ocular case, a 64 year old woman from Belgrade was presented with history of progressive swelling, redness, pain and unpleasant feeling in the right eye conjunctival area. Under ophthalmic examination mass with thin, very active and movable worm wrapped in circles was found. The 8 cm worm was surgically removed under local anesthesia (Figs. 3 and 4). The third case of *Dirofilaria* infection was imported from Tivat, Montenegro in a 22 year old female. The infection was manifested as two weeks increasing subcutaneous nodule (3.5 cm) of the anterior forearm near elbow crease accompanying with pruritus, erythema and pain (Fig. 5). Routine blood tests, including eosinophil count were within normal limits. Abscess was diagnosed, incision was performed and 9 cm worm was extracted.

In the previous two cases *D. repens* was identified according to morphological features in histological sections. All patients provided agreement for participation in this study.

**References**

1. Džamić AM, Čolović IV, Arsić Arsenijević VS, Stepanović S, Boričić I, Džamić Z, Mitrović SM, Rašić DM, I Stefanović, Latković Z, Kranjčić Zec I. Human Dirofilaria repens infection in Serbia. J Helminthol. 2009; 83:129–137.

2. Tasić-Otašević SA, Trenkić-Božinović MS, Gabrielli SV, Genchi C. Canine and human Dirofilaria infection in the Balkan Peninsula. Vet Parasitol. 2015; 209:151–156.

3. Matějů J, Chanová M, Modrý D, Mitková B, Hrazdulová K, Žampacová V, Kolářová L. Dirofilaria repens: emergence of autochthonous human infections in the Czech Republic (case reports). BMC Infect Dis. 2016; 16:171.

4. Kurucz K, Kepner A, Krtinic B, Zana B, Földes F, Bányai K, Oldal M, Jakob F, Kemenesi G. First molecular identification of Dirofilaria spp. (Onchocercidae) in mosquitoes from Serbia. Parasitol Res. 2016, pp 1-4.

### A22 Heartworm disease vectors in Europe – new distribution trends

#### Rodrigo Morchón^1^, Elena Carretón^2^, Paula Josefina Gómez^1^, Alicia Diosdado^1^, Javier González-Miguel^1^

##### ^1^Laboratory of Parasitology, University of Salamanca, Salamanca, 37007, Spain; ^2^Internal Medicine, University of Las Palmas de Gran Canaria, Gran Canaria, 35413, Spain

###### **Correspondence:** Rodrigo Morchón (rmorgar@usal.es)

Several species of (family culicidae) mosquitoes have been identified as vectors of canine and feline cardiopulmonary dirofilariosis in different parts of the world. Its transmission depends mainly on weather conditions, which must be favorable for their development and survival. Europe is a continent where heartworm disease is expanding, but, at the same time, there are very few studies about the transmission vectors. Our aim is to review the current distribution of potential vectors in the European continent, the changes and their possible causes. Various studies had reported several species of mosquitoes infected by *D. immitis* larvae such as Haplotype H1 of *Culex pipiens* in Spain; *Cx. pipens* complex in Italy, Turkey, Germany and Belarus Republic; *Cx. torrentium* in Germany and Belarus; *Cx. theileri* in Madeira (Portugal) and Canary Islands (Spain); *Aedes albopictus*, *Ae. caspius* and *Coquillettidia richiardii* in Italy; *Anopheles maculipennis* in Italia; *Ae. vexans* in Turkey, Slovakia and Czech Republic. On the other hand, a few species of mosquitoes have been described as *D. repens* potential vectors: *An. maculipennis* and *An. algeriensis* in Austria*; An. daciae, Culiseta annulata and An. maculipennis* in Germany; *Ae. vexans* in Slovakia and Germany and *An. claviger* s.l. in Belarus. In these studies have been employed or CO_2_ or animal or human-bait traps [1-7]. Several factors can exert an influence on the emergence or discovery of species or new species to act as vectors, such as the climate change caused by the global warming or the interest in studying this disease in countries when new cases of canine heartworm are diagnosed. The activity of these species is another factor to consider. Mainly, their activity develops in spring and summer and their behavior depends of the different feeding patterns of each specie. For example, *Cx. pipiens*, *Anopheles* spp. are active only during the night while *Ae. Albopictus* predominantly at dawn or during the day*.* More studies and new programs of control of vectors in the current and new endemic countries, and control measures should be carried out to prevent the spreading of this disease.

**References**

1. Bocková E, Iglódyová A, Kočišová A. Potential mosquito (Diptera: Culicidae) vector of *Dirofilaria repens* and *Dirofilaria immitis* in urban areas of Eastern Slovakia. Parasitol Res. 2015; 114: 4487–4492.

2. Kronefeld M, Werner D, Kampen H. PCR identification and distribution of *Anopheles daciae* (Diptera, Culicidae) in Germany. Parasitol Res. 2014; 113: 2079–2086.

3. Montarsi F, Ciocchetta S, Devine G, Ravagnan S, Mutinelli F, Frangipane di Regalbono A, Otranto D, Capelli G. Development of *Dirofilaria immitis* within the mosquito *Aedes* (*Finlaya*) *koreicus*, a new invasive species for Europe. Parasit Vectors. 2015; 8: 177.

4. Morchón R, Carretón E, González-Miguel J, Mellado-Hernández I. Heartworm Disease (*Dirofilaria immitis*) and Their Vectors in Europe - New Distribution Trends. Front Physiol. 2012; 3: 196.

5. Rudolf I, Šebesta O, Mendel J, Betášová L, Bocková E, Jedličková P, Venclíková K, Blažejová H, Šikutová S, Hubálek Z. Zoonotic Dirofilaria repens (Nematoda: Filarioidea) in *Aedes vexans* mosquitoes, Czech Republic. Parasitol Res. 2014; 113: 4663–4667.

6. Sassnau R, Czajka C, Kronefeld M, Werner D, Genchi C, Tannich E, Kampen H. *Dirofilaria repens* and *Dirofilaria immitis* DNA findings in mosquitoes in Germany: temperature data allow autochthonous extrinsic development. Parasitol Res. 2014; 113: 3057–3061.

7. Șuleșco T, Volkova T, Yashkova S, Tomazatos A, von Thien H, Lühken R, Tannich E. Detection of *Dirofilaria repens* and *Dirofilaria immitis* DNA in mosquitoes from Belarus. Parasitol Res. 2016 May 11. [Epub ahead of print]

### A23 Distribution and prevalence of heartworm disease in the canine population in the province of Salamanca (West-central Spain)

#### Alicia Diosdado, Javier González-Miguel, Fernando Simón, Rodrigo Morchón

##### Laboratory of Parasitology, University of Salamanca, Salamanca, 37007, Spain

###### **Correspondence:** Rodrigo Morchón (rmorgar@usal.es)

Since dirofilariosis caused by *Dirofilaria immitis* is a vector-borne disease, its distribution depends on environmental conditions as well as demographic factors and the management of pets by humans [1]. In the province of Salamanca (West-central Spain) the disease is known from many years, appearing in an area with extensive irrigated crops along the river Tormes [2]. Because recent demographic changes have occurred in this area, the present study has been carried out with the aim to monitoring the distribution and prevalence of the disease in the canine population of this area. For that, 191 dogs were analysed through antigen and microfilariae tests and geo-referenced in a map. The general prevalence is 5.76%, although the disease is only present in dogs from municipalities with irrigated crops in which the prevalence is 16.67%. These results indicate that *D. immitis* continues to be present in the province of Salamanca and that it is associated with the presence of irrigations but with a clear decrease in the prevalence. Causes of the prevalence decrease as well as the potential zoonotic risk are discussed.

**References**

1. Simón F, Siles-Lucas M, Morchón R, González-Miguel J, Mellado I, Carretón E, Montoya-Alonso JA. Human and animal dirofilariasis: the emergence of a zoonotic mosaic. Clin Microbiol Rev. 2012; 25: 507–544.

2. Morchón R, Carretón E, González-Miguel J, Mellado-Hernández I. Heartworm Disease (*Dirofilaria immitis*) and Their Vectors in Europe - New Distribution Trends. Front Physiol. 2012; 3:196.

### A24 Diagnostics and therapy of *Dirofilaria immitis* infections in an isolated dog shelter

#### Vladan Panic^1^, Rastko Bekvalac^1^, Ivan Fenjac^1^, Aleksandar Potkonjak^2^, Suzana Otasevic^3^, Sara Savic^4^

##### ^1^Private veterinary practice “Pedigre”, Novi Sad, Serbia; ^2^Department of Veterinary medicine, Faculty of Agriculture, University of Novi Sad, Novi Sad, Serbia; ^3^Department of Microbiology and Immunology, Medical faculty, University of Nis, Public Health Institute Nis, Nis, Serbia; ^4^Scientific Veterinary Institute “Novi Sad, Novi Sad, Serbia

###### **Correspondence:** Sara Savic (sara@niv.ns.ac.rs)

The first published research on *Dirofilaria immitis (D. immitis)* infections in Serbia was in the 1990s, when the first cases were determined in dogs, discovered as a side finding during dissections. So far, after many studies, it can be pointed out that Vojvodina, (Northern Serbia) is an endemic region for dirofilariasis in dogs caused by *D. immitis.* During the period of the last 10 years, prevalence of *D. immitis* infection in dogs went from 7% to 26,9%. Today, clinical symptoms in dogs can be observed, a regular health check-up in dogs is provided by the veterinary service. Herein we report a very high prevalence of *D. immitis* infections in dogs from one dog shelter with a total of 19 dogs near Novi Sad, Vojvodina and good outcomes after Ivermectin therapy. The shelter is situated 20 km away from the city of Novi Sad, close to the river Danube, with a lot of trees and grass surfaces around. Out of a total of 19 dogs, 13 dogs had *D. immitis* infections which were diagnosed at clinical examinations (dogs presented cough and weakness during the regular everyday activities), snap test (SNAP 4DX Idexx) and by Knott test for detection of microfilariae in peripheral blood. In all infected dogs, therapy was started with Ivermectin, with a dose of 0.6 mg/kg per body mass every week for 4 weeks, then every two weeks, followed by once per month. The monitoring of therapy effectiveness was performed every month in all of the dogs due clinical examination and Knott testing. After therapeutic procedure, microfilariae were not detected in blood of all cured dogs. Therapy with Ivermectin and Knott test were repeated for the next 6 months and there were no parasitological positive findings of dirofilariosis. In addition, eight months after the first therapy was given to all of the dogs, parasitological and clinical examinations showed that all dogs were without clinical symptoms and using Knott test microfilariae were not found in blood of examined dogs.

### A25 Detection of *Dirofilaria* spp. in dogs from Greece: Preliminary results

#### Elias Papadopoulos^1^, Athanasios Angelou^1^, Eleftherios Gallidis^1^, Kyriakos Spanoudis^1^, Roland Schaper^2^, Ramaswamy Chandrashekar^3^

##### ^1^School of Veterinary Medicine, Aristotle University, Thessaloniki, 541 24, Greece; ^2^Bayer Animal Health GmbH, Leverkusen, 51368, Germany; ^3^IDEXX Laboratories, Inc., Westbrook, Maine 04092, USA

###### **Correspondence:** Elias Papadopoulos (eliaspap@vet.auth.gr)

Dirofilariosis is an important parasitic disease of dogs, cats and wild carnivores worldwide. It is among the most common canine vector-borne disease and represents a serious threat to both animal and public health [1]. Greece is a typical Mediterranean country with reported cases of Dirofilaria-infected animals [2] and has favourable climatic conditions for mosquitoes, including new invasive species. The aim of this study was to investigate the presence of *Dirofilaria* spp. in clinically healthy dogs and to create a prevalence map including all geographic parts of the country. Blood samples were collected from a total of 276 dogs. They were animals of different breeds, both indoor and outdoor and used for different purposes (hunting, guarding, pets, shepherds, stray etc.). All samples were tested with the SNAP® 4Dx® Plus Test to detect *Dirofilaria immitis* antigen. In addition, samples were examined by Knott test to identify microfilariae of *D. immitis* and *D. repens*. Additional data were collected in order to identify potential risk factors. Thirty-two *D. immitis* antigen positive samples (11.6%) were detected by serology and19 of 32 were amicrofilariaemic. *D. repens* microfilariae were identified in 4 (1.4%) dogs. Infected dogs were originating significantly more from Northern than Southern parts of Greece (p < 0.01). Also, dogs at higher risk were the ones spending more time or activity outside the house (i.e. hunting) and with minimum preventive antiparasitic administration. These results revealed a high occurrence of *Dirofilaria* spp. in clinically healthy dogs in Greece and highlight the need to maintain a comprehensive and regular prophylaxis to reduce the contact between dogs and mosquito vectors. Furthermore, the findings of this study confirm that clinically healthy dogs need to be routinely screened for this parasite, as early diagnosis may be an important component of successful treatment and public health protection.

The study was funded by Bayer Animal Health GmbH.

**References**

1. Miliaras D, Meditskou S, Kelekis A, Papachristos I. Human pulmonary Dirofilariasis: one more case in Greece suggests that *Dirofilaria* is a rather common cause of coin lesions in the lungs in endemic areas of Europe. Int J Immunopathol Pharmacol. 2010; 23: 345–348.

2. Polizopoulou ZS, Koutinas AF, Saridomichelakis MN, Patsikas MN, Leontidis LS, Roubies NA, Desiris AK. Clinical and laboratory observations in 91 dogs infected with *Dirofilaria immitis* in northern Greece. Vet Rec. 2000; 146: 466–469.

### A26 Subjective and objective assessment of radiographic findings in dogs with heartworm disease

#### Ljubica Spasojevic Kosic^1^, Vesna Lalosevic^1^, Aleksandar Naglic^2^, Stanislav Simin^1^, Ljiljana Kuruca^1^, Aleksandar Spasovic^3^

##### ^1^Department of Veterinary Medicine, Faculty of Agriculture, University of Novi Sad, Novi Sad, 21000, Serbia; ^2^JKP ”Zoohigijena i veterina”, Novi Sad, 21000, Serbia; ^3^PVA ”Mama”, Belgrade, 11000, Serbia

###### **Correspondence:** Ljubica Spasojevic Kosic (ljubicask@polj.uns.ac.rs, ljubica.spasojevic@gmail.com)

Thoracic radiography is a very important diagnostic procedure for establishing a diagnosis of the heartworm disease (HWD). It enables an insight into the morphology of a lung field and cardiac silhouette. Radiographic changes associated with HWD can be assessed both subjectively and objectively. The aim of this work is to score subjective changes associated with canine heartworm disease in order to make them more comparable and useful for clinicians. Within objective measurements, in addition to the determination of a heart size, sizes of relevant blood vessels were determined according to the vertebral heart scale (VHS) system. Thoracic radiographs from 20 dogs with natural heartworm disease were measured. Both recumbent lateral (LL) and dorsoventral (DV) radiographs were available from 16 dogs whilst lateral recumbent radiographs were available from 4 dogs. The diagnosis of the heartworm infestation was established according to the results of wet blood smears, modified Knott test [1] and heartworm antigen test. Radiographs of each dog were assessed subjectively (vascular, alveolar and interstitial pattern and right-sided cardiomegaly) [2] and objectively (VHS) [3, 4]. A stage of heartworm disease was determined for each dog. Results were statistically analyzed and presented as percentages (qualitative variables) and mean ± standard deviation (SD) (quantitative variables). In this descriptive retrospective study we defined an incidence of each radiographic change and scored them, and calculated sizes of a heart and blood vessels relevant to the HWD among examined dogs. The most common radiographic changes subjectively assessed were increased sternal contact (95%) and rounding of the cranial border (90% of dogs). Scores for subjective assessment of radiographic findings in examined dogs were in the range 2/9 to 5/9 for cardiomegaly and 1/12 to 6/12 for lung pattern. Average heart sizes measured in LL and DV radiographs were 10.75 ± 0.78v and 11.04 ± 0.42v, respectively. Measurements of relevant blood vessels were as follows: vena cava caudalis 0.83 ± 0.10v, right cranial lobar artery 0.31 ± 0.08v and right caudal lobar artery 0.96 ± 0.42v. Further studies are needed to compare these results with results of dogs without HWD in order to define the most important changes that could be used as a diagnostic or prognostic tool. Objectivity in the assessment of the radiographs of dogs with HWD is possible to achieve by scoring the findings and using objective radiographic measurement.

This work is part of the research done in the project TR31084 granted by the Serbian Ministry of Education and Science.

**References**

1. Bazzocchi C, Mortarino M, Grandi G, Kramer LH, Genchi C, Bandi C, Genchi M, Sacchi L, McCall JW. Combined ivermectin and doxycycline treatment has microfilaricidal and adulticidal activity against Dirofilaria immitis in experimantally infected dogs. Inetern J Parasiotol. 2008; 38: 1401–1410.

2. Herrtage M, Denis R. The Thorax. In: Lee R, editor. Manual of Small Animal Diagnostic Imaging. Second edition, Quedgeley: BSAVA; 1995.p 43-67.

3. Buchanan JW, Bückeler J. Vertebral scale system to measure canine heart size in radiographs. J Am Vet Med Assoc. 1995; 206: 194–199.

4. Spasojevic Kosic Lj, Krstic N, Trailovic RD. Comparison of three methods of measuring vertebral heart size in German Shepherd dogs. Acta vet. 2007; 57: 133–141.

### A27 Occurrence and taxonomical classification of microfilariae in blood samples from canine blood donors localized in south-eastern Poland

#### Tomczuk Krzysztof^1^, Szczepaniak Klaudiusz^1^, Grzybek Maciek^1^, Andrzej Junkuszew^2^, Paulina Dudko^2^, Pantchev Nikola^3^, Stefaniak Marzena^4^, Iwanicki Ryszard^4^

##### ^1^Department of Parasitology and Invasive Diseases, University of Life Sciences, Lublin, ul. Akademicka 12, 20-950 Lublin, Poland; ^2^Faculty of Biology and Animal Breeding Department of Small Ruminants Breeding and Agriculture Advisory, University of Life Sciences In Lublin ul. Akademicka 13 20-950 Lublin, Poland; ^3^IDEXX Laboratories, 71636 Ludwigsburg, Germany; ^4^Lubelskie Centrum Małych Zwierząt, ul. Stefczyka 11, 20-151 Lublin, Poland

###### **Correspondence:** Tomczuk Krzysztof (krzysztof.tomczuk@up.lublin.pl)

Blood transfusions are routinely performed in small animal veterinary hospitals. However, in many practices a screening of blood donors for canine vector-borne diseases (CVBDs) is not a mandatory procedure. So far dogs have been not tested for the occurrence of microfilariae in most Polish canine blood banks, which indicates lack of available data regarding microfilariosis among canine blood donors. The survey was carried out in the second half of year – between May and December 2015 what corresponds to the highest levels of microfilariae per ml observed in peripheral blood of dogs from Central and Eastern Europe. A total of 350 blood samples from healthy dogs - blood donors, were analyzed using microscopic and biomolecular methods. Microfilaraemic samples were further analyzed by standard PCR methods. Circulating microfilariae were detected in fresh smear in 20 samples with prevalence of 5.7% (3.6-9.0). PCR analyzed revealed that, in total 16 out of 20 samples were positive for *D. repens* while 4 samples were negative. Other filarial species (*D. immitis, Acanthocheilonema reconditum, A. dipetalonema dracunculoides*) occurring in Europe were not detected in the analyzed material. Canine dirofilariosis has been spreading during the last years in Central Europe countries [1]. *D. repens* is a dominant causative agent of canine microfilariosis in Poland [2], which was confirmed in our study. Currently a cross-serological survey also revealed a circulating antigen of *D. immitis* in these geographical areas. In Poland 0.015% dogs were positive for circulating antibodies *D. immitis* [3]. Despite the fact that dogs cannot infect *Dirofilaria* spp. via blood transfusion, the risk of spreading the reservoir invasion and possible immune reactions of the host (blood recipient) indicate that screening tests for dirofilariosis are essential.

**References**

1 Miterpáková M, Iglódyová A, Čabanová V, Stloukal E, Miklisová D. Canine dirofilariosis endemic in Central Europe-10 years of epidemiological study in Slovakia. Parasitol Res. 2016; 115: 2389–2395.

2 Demiaszkiewicz AW, Polańczyk G, Osińska B, Pyziel AM, Kuligowska I, Lachowicz J, Sikorski A. The prevalence and distribution of Dirofilaria repens in dogs in the Mazovian Province of central-eastern Poland. Ann Agric Environ Med. 2014; 21: 701–704.

3 Krämer F, Schaper R, Schunack B, Połozowski A, Piekarska J, Szwedko A, Jodies R, Kowalska D, Schüpbach D, Pantchev N. Serological detection of Anaplasma phagocytophilum, Borrelia burgdorferi sensu lato and Ehrlichia canis antibodies and Dirofilaria immitis antigen in a countrywide survey in dogs in Poland. Parasitol Res. 2014; 113: 3229–3239.

### A28 Filarioid helminths in mosquitoes from the Danube Delta/Romania and the analysis of these vectors for potential vector competence

#### Victoria Wimmer^1^, Angela Monica Ionică^2^, Carina Zittra^1^, Natascha Leitner^1^, Jan Votýpka^3^, David Modrý^4,5^, Andrei Daniel Mihalca^2^, Hans-Peter Fuehrer^1^

##### ^1^Department of Pathobiology, Institute of Parasitology, University of Veterinary Medicine Vienna, 1210 Vienna, Austria; ^2^Department of Parasitology and Parasitic Diseases, Faculty of Veterinary Medicine, University of Agricultural Sciences and Veterinary Medicine, Calea Mănăştur 3-5, Cluj-Napoca, Romania; ^3^Department of Parasitology, Faculty of Sciences, Charles University, Viničná 7, 12844 Prague, Czech Republic; ^4^Department of Pathology and Parasitology, Faculty of Veterinary Medicine, University of Veterinary and Pharmaceutical Sciences, Palackého tr. 1946/1, 612 42 Brno, Czech Republic; ^5^Biology Centre, Institute of Parasitology, Czech Academy of Sciences, Branisovska 31, 370 05 České Budějovice, Czech Republic

###### **Correspondence:** Hans-Peter Fuehrer (hans-peter.fuehrer@vetmeduni.ac.at)

In the past decades both *Dirofilaria immitis* and *D. repens* have spread from historically endemic areas to central and eastern European countries. Several studies have shown that *Dirofilaria* species are present in the southern and south-eastern areas of Romania [1]. However, information about the vectors in the Danube Delta and their vector competence is lacking. In July 2015 more than 5,000 mosquitoes were collected in the Danube Delta in Romania at various locations (including mosquito traps next to a dog infected with both *D. immits* and *D. repens*). Mosquitoes were classified to species-level using the key after Becker et.al. [2]. In one part of the study specified mosquitoes were pooled (up to 25 individuals per day/trap/mosquito species). DNA was extracted and the samples were screened for filarioid helminths using conventional PCRs. For the second part of the study 300 specified mosquito individuals caught at the trap next to a microfilariaemic dog positive for *D. immitis* and *D. repens* were segregated into head/thorax and abdomen prior to DNA extraction. Each thorax/head and abdomen was screened for the presence of filarioid DNA separately. All positive PCR products were further analysed by sequencing.

Mosquitoes were sampled within the training school of WG1 under the frame of EurNegVec COST Action TD1303. Parts of this study were funded by the ERA-Net BiodivERsA, with the national funders FWF I-1437, ANR-13-EBID-0007-01 and DFG BiodivERsA KL 2087/6-1 as part of the 2012-13 BiodivERsA call for research proposals.

**References**

1. Ionică AM, Matei IA, Mircean V, Dumitrache MO, D'Amico G, Győrke A, Pantchev N, Annoscia G, Albrechtová K, Otranto D, Modrý D, Mihalca AD. (2015) Current surveys on the prevalence and distribution of *Dirofilaria* spp. and *Acanthocheilonema reconditum* infections in dogs in Romania. Parasitol Res. 114(3):975-82. doi: 10.1007/s00436-014-4263-4.

2. Becker N, Petrić D, Zgomba M, Boase C, Madon M, Dahl C, Kaiser A. Mosquitoes and their control. Berlin: Springer; 2010.

## 4^TH^ BAYER ANGIOSTRONGYLOSIS FORUM 2016

### A29 *Angiostrongylus vasorum* – what’s new?

#### Manuela Schnyder (manuela.schnyder@uzh.ch)

##### Institute of Parasitology, University of Zurich, 8057 Zurich, Switzerland

The increasing number of publications since the turn of the millennium mirrors the growing interest in *Angiostrongylus vasorum*. The most recent works have focussed on various aspects of the infection. First of all, the expansion of *A. vasorum* in dogs and in wildlife in Europe seems to persist. New reports include cases in dogs from Belgium, Portugal, Bulgaria and Slovakia, all surrounded by countries where *A. vasorum* had previously been observed. In parallel, studies in wildlife have confirmed that foxes represent the most important reservoir, with prevalences over 70%.

Recent epidemiological studies in foxes and dogs showed that annual precipitation and temperature influenced the distribution of *A. vasorum*, and that in the Alps, altitudes above 700 m asl represent a limiting factor for parasite transmission. Field studies illustrated the variability of spatial distribution and the variability of the slug fauna acting as intermediate hosts, which was suggested to explain the clumpy distribution of *A. vasorum*. An additional confounder may be represented by birds: in addition to previously described frogs, experimental studies have shown that chicken (and therefore potentially other bird species), may also act as paratenic hosts.

In dogs, the classical larval detection in faeces is frequently complemented with PCR performed on different substrates, including bronchoalveolar fluid. Comparisons performed between coproscopic, biomolecular and serological methods testify to the high performance of serological methods. A commercially available test kit for *A. vasorum* antigen detection allows the diagnosis of canine angiostrongylosis within 15 minutes. It also proved highly sensitive when analysing cardiopulmonary tissue fluid of foxes. Last but not least*,* the broad variety of clinical signs associated with *A. vasorum* infection accounts for an excellent camouflaging of the disease, including manifestations in the eyes, neurological disorders, bleeding from various surfaces or internally or even by nematode dermatitis, hepatic abnormalities or concurrent infections with the heartworm *Dirofilaria immitis.* In clinical patients with respiratory distress the occurrence of pulmonary hypertension was proposed as a negative predictor of survival to the infection Importantly, bleeding seems to occur in up to one third of clinical cases, however results of tests evaluating the coagulation system are not fully consistent and the reasons behind the impaired coagulation are still debated.

In conclusion, the clinical diagnosis of angiostrongylosis represents a challenge, therefore disease awareness is pivotal. Moreover, a wide range of open questions remain to be addressed.

### A30 *Angiostrongylus vasorum* in its intermediate hosts: an epidemiological survey in Germany

#### Malin Lange^1^, Felipe Penagos^1^, Carlos Hermosilla^1^, Roland Schaper^2^, Anja Taubert^1^

##### ^1^Institute of Parasitology, Justus Liebig University, Gießen, Germany, 35392; ^2^Bayer Health GmbH, Leverkusen, Germany, 51368

###### **Correspondence:** Malin Lange (malin.k.lange@vetmed.uni-giessen.de)

Infections with the French Heartworm *Angiostrongylus vasorum* represent neglected diseases of dogs in Germany. Due to the localization of *A. vasorum* in the right heart and pulmonary artery this parasite causes a multi-factorial disease being represented by general, respiratory, circulatory, bleeding and neurological disorders that occasionally lead to death. Recent European surveys indicate that this parasite is spreading in Europe. Actual data on prevalences in dogs and foxes (acting as reservoir hosts) reveal several endemic foci in Germany. The life cycle of *A. vasorum* is obligatory linked to an intermediate host being represented by a wide range of slugs and snails. Given that actual data on *A. vasorum* infections in intermediate hosts are missing for Germany, we here conducted an epidemiological survey on slugs in selected regions of Hesse and Rhineland-Palatinate. To account for seasonal variations slugs were collected throughout the season in spring, summer, autumn and winter in four different areas (two spots for Hesse and Rhineland-Palatinate, each) that were previously proven to be hyperendemic for *A. vasorum* fox infections. Thus, a total of 2701 slugs were collected and examined for lungworm larvae using the techniques of artificial digestion and microscopy. The confirmation of the lungworm species will be made by specific PCRs. Preliminary data revealed a total *A. vasorum* prevalence of 4.6% in slugs based on microscopic analyses. The number of *A. vasorum* larvae per slug varied considerably (1-546 larvae per specimen). Considering the different sampling areas, some hotspots with relatively high *A. vasorum* prevalences in slugs (up to 10%) were identified. *A vasorum* prevalences varied with the season since highest prevalences were detected in summer (9.1%), whilst the lowest number of infected slugs was found in winter (0.8%). Besides *A. vasorum*, we additionally detected other lungworm larvae in slug samples: *Crenosoma vulpis/striatum* (lungworm of dog/hedgehog, 2.2%) and *Aelurostrongylus abstrusus* (feline lungworm, 0.2%). Overall, the current data demonstrate that dogs are at a permanent risk for *A. vasorum* infections (even in winter) when living in the investigated areas.

### A31 Seroprevalence of *Angiostrongylus vasorum* in Swedish dogs: a national survey

#### Giulio Grandi^1^, Eva Osterman-Lind^1^, Roland Schaper^2^, Ulrika Forshell^3^, Manuela Schnyder^4^

##### ^1^Department of Microbiology, National Veterinary Institute, Uppsala, 75189, Sweden; ^2^Bayer Animal Health GmbH, Leverkusen, Germany; ^3^Bayer HealthCare - Animal Health, Copenhagen, Denmark; ^4^Institute of Parasitology – University of Zurich, Zurich, Switzerland

###### **Correspondence:** Giulio Grandi (giulio.grandi@sva.se)

*Angiostrongylus vasorum*, the French heartworm, is a parasite of dogs described in several parts of the world, including continental Europe and the British Isles [1]. Regarding parasite occurrence in Scandinavia, endemic foci are widely present in Denmark and recently the parasite has been found in foxes in Norway [2]. In Sweden the parasite was first identified in 2003 on the island of Sydkoster (Västra Götaland County) [3]. Since then Swedish sporadic endemic cases of *A. vasorum* were diagnosed through positive canine faecal samples every year since 2011. A progressively increasing number of faecal samples has been submitted to SVA (National Veterinary Institute, Uppsala), however the prevalence in dogs appears to be quite low. A large-scale collection of canine serum samples was planned in order to identify the presence and distribution of *A. vasorum* in Sweden using more sensitive methods, i.e. serological methods able to detect parasite antigens and antibodies developed against the parasite. In this first large scale survey, 3886 sera from pet dogs were collected from the Clinical Chemistry Laboratory of the University Animal Hospital (UDS-SLU, Uppsala) as well as from SVA and 3309 (85% of 3886) have been tested until now by an ELISA for the detection of circulating antigen of *A. vasorum* and by a separate ELISA detecting specific antibodies against the parasite. Among the analysed samples a total of 0.39% (n = 13, 95% Confidence Intervals, CI: 0.21-0.67%) of the animals were positive in both ELISAs, while 0.70% (n = 12, CI: 0.44-1.04%) of the tested dogs were antigen-positive only and 1.48% (n = 49, CI: 1.48-1.10%) were positive for specific antibodies only. These preliminary results confirm that *A. vasorum* is established in Sweden with a prevalence comparable to other European countries. Definitive results from ongoing analyses will provide a deeper insight on the dissemination of the parasite over the country.

**References**

1. Elsheikha HM, Holmes SA, Wright I, Morgan ER, Lacher DW. Recent advances in the epidemiology, clinical and diagnostic features, and control of canine cardio-pulmonary angiostrongylosis. Vet Res. 2014; 45:92.

2. VV AA. Fransk hjerteorm påvist for første gang i Norge. Website of the Norwegian Veterinary Institute, http://vetinst.prod1.seeds.no/eng, accessed 14th of April, 2016.

3. Åblad B, Christensson D, Osterman Lind E, Ågren E, Mörner T. *Angiostrongylus vasorum* etablerade i Sverige. Svensk Veterinär Tidning. 2003; 55:11–15.

### A32 Geographical distribution of metastrongylid nematodes *Angiostrongylus vasorum* and *Crenosoma vulpis* in Slovak wildlife - preliminary study

#### Viktória Čabanová, Zuzana Hurníková, Martina Miterpáková

##### Institute of Parasitology, Slovak Academy of Sciences, Košice, 040 01, Slovakia

###### **Correspondence:** Martina Miterpáková (miterpak@saske.sk)

*Angiostrongylus vasorum* and *Crenosoma vulpis* are important lungworms infecting dogs and wild canids, and their incidence is increasing worldwide. In Europe, red fox (*Vuples vulpes*) is considered as major reservoir host of these species. With regard to successful anti-rabies vaccination programmes and their urbanisation, red foxes represent significant infection risk for dogs. Despite it, data on the occurrence of these parasites in fox populations are very scant in a lot of European countries. In Slovakia, *A. vasorum* in dogs was for the first time reported in 2013 and then in 2014 [1, 2]. Consequential serological survey confirmed circulating *A. vasorum* antigen or the parasite-specific antibodies in 6.22% of dogs investigated [3]. *A. vasorum* was not previously reported in Slovak red foxes. On the other hand, *C. vulpis* was noticed in 1960ties and 1980ies in red foxes from Tatra National Park, Northern Slovakia, but its distribution and prevalence rate has never been formally surveyed. Therefore, the aim of the present study was to uncover real occurrence of *A. vasorum* and *C. vulpis* in fox population throughout Slovakia and estimate the risk of infection for dogs. Between September 2015 and April 2016 faecal samples of 420 red foxes were examined using flotation technique with zinc sulphate and Baermann migration method. The first stage larvae were determined by morphometric and morphological characteristics. Of 420 red foxes, 80 (19.05%) were positive for *C. vulpis* and 25 (5.95%) for *A. vasorum*. Only one fox showed dual infection with the both species. Geographic information system was used to map the spatial distribution of infected foxes. In conclusion, it should be said, it is the first monitoring of *A. vasorum* and *C. vulpis* in Slovak foxes and the data obtained will serve for any future epidemiological researches.

**Acknowledgement**

The research was supported by the Slovak Grant Agency VEGA, projects No. 2/0018/16.

**References**

1. Hurníková Z, Miterpáková M, Mandelík R. First autochthonous case of canine *Angiostrongylus vasorum* in Slovakia. Parasitol Res, 2013; 112: 3505–3508.

2. Miterpáková M, Hurníková Z, Zalewski A.P. The first clinically manifested case of angiostrongylosis in a dog in Slovakia. Acta Parasitol, 2014; 59: 661–665.

3. Miterpáková M, Schnyder M, Schaper R, Hurníková Z, Čabanová V. Serological survey for canine angiostrongylosis in Slovakia. Helminthologia, 2015; 52: 205–210.

### A33 Baermann fecal examination survey of dogs showing signs of respiratory disease in Ontario, Canada

#### Gary Conboy^1^, Nicole Murphy^1^, Tamara Hofstede^2^

##### ^1^Department of Pathology and Microbiology, Atlantic Veterinary College, Charlottetown, Prince Edward Island, C1A 4P3, Canada; ^2^Animal Health Bayer Inc, Mississauga, Ontario, L4W 5R6, Canada

###### **Correspondence:** Gary Conboy (conboy@upei.ca)

Canine respiratory disease due to helminth infection is considered infrequent. Diagnosis is challenging due to poor detection sensitivity of fecal flotation for most species of lungworms. Along with an over-reliance in clinical practice on fecal flotation for detection of parasitism, this leads to the potential for under-diagnosis of lungworms. A further complication is the sporadic fecal larval shedding patterns typical of metastrongyloid infections. Fecal samples (3 consecutive day collections) from dogs showing signs of respiratory disease were examined for the presence of lungworm first-stage larvae (L1) or eggs using the Baermann technique and zincsulfate centrifugal flotation from October 2014 to May 2016. Afrebrile dogs showing signs of respiratory disease (mainly chronic cough) that had not received an anthelmintic (excepting pyrantel or selamectin) within the last 60 days were included in the study. Baermann examinations were done on a 12-gram composite sample (4 grams of feces from each of the 3 collection days) and a 12-gram sample (day 3 collection) for each dog. Larval counts (L1/gram feces = LPG) were done on each of the 3 day collection samples if larvae were detected on either the composite or day 3 sample. Helminths known to cause respiratory disease were detected in 6.9% (22/317) of the samples examined. Duration of clinical signs prior to diagnosis ranged from 14 – 210 days. First-stage larvae of *Crenosoma vulpis* (4.7%; 15/317), *Strongyloides stercoralis* (0.6%; 2/317), *Filaroides hirthi/Oslerus osleri* (0.3%; 1/317) and *Aelurostrongylus abstrusus* (0.3%; 1/317) were detected on Baermann examination. Detection of *A. abstrusus* L1 in the one dog was considered a spurious finding. Eggs of *Paragonimus kellicotti* (0.6%; 2/317) and *Eucoleus boehmi* (0.3%; 1/317) were detected on centrifugal flotation. All of the *C. vulpis* infections were detected from October to May with nearly half occurring in March. Baermann examination of the 3-day composite sample detected 86.7% (13/15) of the *C. vulpis* infections compared to 73.3% (11/15) detection by examination of a single (day 3) sample. Larval shedding levels ranged from 0 – 455 LPG (Mn = 22.2 LPG); only 2 dogs shed more than 20 LPG. Lungworm infection should be considered as a possible cause in any case of respiratory disease in dogs in eastern Canada (and likely elsewhere). Three daily Baermann fecal examinations had greater *C. vulpis* detection sensitivity than a 3-day collection composite and both were superior to examination of a single day collection sample.

### A34 Lungworms in Germany 2003 - 2015 - a true increase?

#### Dieter Barutzki^1^, Viktor Dyachenko^1^, Roland Schaper^2^

##### ^1^Veterinary Laboratory Freiburg, Freiburg, Germany, 79108; ^2^Bayer Animal Health GmbH, Leverkusen, Germany, 51368

###### **Correspondence:** Dieter Barutzki (barutzki@labor-freiburg.de)

In recent years, infections with *Angiostrongylus vasorum* in dogs have increasingly been reported in European countries. For some time occurrence and distribution of *A. vasorum* seemed to be largely confined in isolated endemic foci. New reports of cases in dogs in endemic areas and data of post mortem surveys of foxes in areas previously believed to be free from infections suggest that *A. vasorum* has increased in prevalence and is spreading geographically within Europe. In Germany only few epidemiological studies have been performed and data on changes in the lungworm distribution in dogs in Germany are lacking. The aim of this study was to present actual data on occurrence and regional geographical distribution of *A. vasorum* and

*C. vulpis* in dogs in Germany and to analyse these data in terms of evidence for geographically spreading of lungworms in Germany. In a retrospective study, the results of parasitological examinations of faecal samples, which had been submitted to the Veterinary Laboratory Freiburg, from 54,934 dogs between 2003 and 2015 in Germany were analysed. All faecal samples were obtained from privately owned dogs presented to local veterinary surgeons from all parts of Germany for mostly unknown clinical problems, routine examination and animal vaccination or general health check. All specimens were tested by a standardised flotation method with a saturated salt solution and examined by Baermann funnel technique to detect first-stage larvae (L1) of lungworms. The collected data were analysed by a geographic information system (GIS) using the programme RegioGraph 10 (GfK GeoMarketing, Bruchsal) to visualise the regional distribution of *A. vasorum* and *C. vulpis*. Rates of infection with *A. vasorum* and *C. vulpis* and their geographical distribution were analysed and proved statistically. From 2003 to 2015 *A. vasorum* and *C. vulpis* were detected in 477 (0.9%) and 248 (0.5%) of 54,934 examined dog samples, respectively. The percentage of *A. vasorum* positive dogs increased 2004 - 2006, 2007 - 2009, 2010 - 2012, and 2013 - 2015 from 0.1%, 0.7%, 0.9% to 1.8%, respectively. In 2014 and 2015 the rates of infection with *A. vasorum* were significantly higher (p < 0.05) compared to each year in 2003 - 2007 and in 2003 - 2010, respectively. There were no statistically significant differences between the rates of infection with *C. vulpis* of every year in 2003 - 2015. Most of the infected dogs with *A. vasorum* and *C. vulpis* were found in south-western Germany. Clusters of infections with *A. vasorum* were located in Baden-Wuerttemberg, Saarland, North Rhine-Westphalia and Rhineland-Palatinate. Our data and the expanding range from which canine cases are reported are consistent with the hypothesis that *A. vasorum* is spreading.

### A35 A coprological and serological survey on *Angiostrongylus vasorum* in Southern Belgium

#### Laetitia Lempereur^1^, Ludovic Martinelle^2^, Calixte Bayrou^3^, Françoise Marechal^1^, Anne-Catherine Dalemans^3^, Bertrand J Losson^1,4^

##### ^1^University of Liège, Faculty of Veterinary Medicine, Laboratory of Parasitology and Parasitic Diseases, Liège, Belgium; ^2^University of Liège, Faculty of Veterinary Medicine, Experimental Station CARE – FePex, Center for Fundamental and Applied Research for Animal and Health (FARAH), Liege, Belgium; ^3^University of Liège, Faculty of Veterinary Medicine, Laboratory of Pathology, Liège, Belgium; ^4^Bayer Health Care, Diegem, Belgium

###### **Correspondence:** Bertrand J Losson (blosson@ulg.ac.be)

Despite the fact that epidemiological models indicate that Belgium has a favourable climate for the completion of *A. vasorum* life cycle [1], the parasite was not recorded in this country until 2013 [2]. The aim of the present study was to gain additional information on the distribution and prevalence of *A. vasorum* infection in dogs through the combined used of in-house detection of circulating specific antigen and coprology. The survey was conducted from November 2014 until February 2016. Seventeen practices were selected across Southern Belgium. Samples were collected from dogs belonging to two populations: a first random dog population (called « control, thereafter) presented for unrelated conditions whereas the second population included dogs showing clinical signs compatible with angiostrongylosis. These two populations were selected based on the absence of travel history outside Belgium during the 3 previous months. Blood samples were collected and an in-clinic serological test detecting *A. vasorum* circulating Ag (Angio Detect™, IDEXX) was used for initial screening. Stools were collected on 3 consecutive days from dogs with a positive serological screening and examined with the Baermann technique [3]. This was not always possible and in some cases stools were obtained only once or twice. A total of 979 dogs were enrolled. Seven hundred fifty-seven were included in the control group whereas 222 dogs had clinical signs compatible with angiostrongylosis. The distribution of samples according to the different tests is given in Table 1. Forty-six dogs out of 979 (4.7%) had *A. vasorum* circulating antigen. However, there was a marked difference between the two populations (3.6 and 8.6% in control and symptomatic dogs respectively). Stools were obtained from 47 dogs (25 and 22 in control and symptomatic dogs respectively). Interestingly larvae of *Crenosoma vulpis* were detected in 1 control and 8 symptomatic dogs respectively. In the latter group one dog was found seropositive for *A. vasorum* but only *C. vulpis* larvae were found via the Baermann technique. All seropositive and symptomatic dogs (n = 19) exhibited cardio-pulmonary symptoms. In conclusion this seroepidemiological study demonstrated a fairly high seroprevalence in Southern Belgium for *A. vasorum*. The Angio detect™ IDEXX was found to be highly suitable in this context as the sampling, preservation and examination of stools were difficult and somewhat unreliable in the field. However, coproscopy remains a useful tool in dogs infected for less than 9 weeks and for the identification of other canine lung nematodes such as *Crenosoma vulpis*.

**References**

1. Jolly S, Poncelet L, Lempereur L, Caron Y, Bayrou C, Cassart D, Grimm F, Losson B. First report of a fatal autochtonous canine *Angiostrongylus vasorum* infection in Belgium. Parasitol. Int. 2014, 64: 97–99.

2. Morgan E., Jefferies R., Krajewski M., Ward P., Shaw S. Canine pulmonary angiostronylosis. The influence of climate on parasite distribution. Parasitol. Internat., 2009, 58: 406–410.

3. Elsheikha H, Holmes S, Wright I., Morgan E, Lacher D. Recent advances in the epidemiology, clinical and diagnostic features, and control of canine cardio-pulmonary angiostrongylosis. Vet. Res., 2014, 45: 92–103.

**Table 1:** Distribution of samples according to the different tests (serology versus coprology)

		Control dogs (n=757) (%)	Symptomatic dogs (n=222) (%)	Total (n=979) (%)
Angio detect™ IDEXX +		27 (3.6)	19 (8.6)	46 (4.7)
Angio detect™ IDEXX + and Baermann +	L1 *A*. vasorum L1 *C*. vulpis L1 *A*. vasorum and *C*. vulpis	7 (1.0)	6 (2.7) 1 (0.45) 2 (0.9)	13 (1.3) 1 (0.1) 2 (0.2)
Angio detect™ IDEXX + and Baermann –		17	8	25
Angio detect™ IDEXX + and Baermann not performed		3	2	5
Angio detect™ IDEXX – and Baermann +	L1 *A*. vasorum L1 *C*. vulpis	1	4 1	5 1

### A36 Risk factors for natural infection with *Angiostrongylus vasorum* in dogs: 100 cases (2003-2009)

#### Hany M. Elsheikha, Sarah B Holmes

##### University of Nottingham, Loughborough, Leicestershire, LE12 5RD, UK

###### **Correspondence:** Hany M. Elsheikha (hany.elsheikha@nottingham.ac.uk)

Canine angiostrongylosis is a snail-borne parasitic infection caused by the nematode *Angiostrongylus vasorum*. The nematode has a complex life cycle, potentially involving an array of intermediate, paratenic and definitive hosts. The global geographical boundaries of infection are spreading to encompass areas where infection was previously uncommon, thus presenting a growing threat to the canid population. Clinical signs of *A. vasorum* infection in dogs are serious and can potentially lead to death. This retrospective study was conducted to evaluate trends in demographic factors and clinical presentation of 100 dogs with *A. vasorum*. Variables analyzed included dog age, breed, gender and frequency of clinical signs. A significant relationship was detected between young age and *A. vasorum* infection, supporting the hypothesis that age-related differences exist in response to *A. vasorum* infection. Gender was not identified as a significant risk factor associated with *A. vasorum* infection in dogs. The breeds that have the highest prevalence of angiostrongylosis were Cocker Spaniels and Labradors, with 13% and 12% of dogs respectively. Significant association was made between *A. vasorum* infection and dogs presenting with cough, coagulopathy, vomiting/diarrhoea and/or lethargy (*p* < 0.05). Taking account of these classical clinical presentations gundogs are highly likely to present with all signs, whereas terriers are presented less often with a coagulopathy (9%) than with the other signs, and hounds are less often with a cough (2%). These findings are important because they provide clues regarding the risk of infection to an individual dog, facilitate improved recognition of infection based upon clinical presentation, and should allow implementation of preventative strategies to combat infection.

**References**

1. CHAPMAN PS, BOAG AK, GUITIAN J, BOSWOOD, A. *Angiostrongylus vasorum* infection in 23 dogs (1999 - 2002). J Small Anim Pract 2004, 45: 435–440.

2. KOCH J, WILLESEN JL. Canine pulmonary angiostrongylosis: An update. The Veterinary J 2009;179, 348–359

3. MORGAN ER, SHAW SE, BRENNAN SF, De WAAL TD, JONES BR, MULCAHY G. *Angiostrongylus vasorum*: a real heartbreaker. Trends Parasitol 2005, 21: 49–51.

4. TAUBERT A, PANTCHEV N, VRHOVEC MG, BAUER C, HERMOSILLA C. Lungworm infections (*Angiostrongylus vasorum*, *Crenosoma vulpis*, *Aelurostrongylus abstrusus*) in dogs and cats in Germany and Denmark in 2003-2007. Vet Parasitol 2009, 159: 175–180.

### A37 Prevalence of lungworms in Swiss red foxes and evaluation of serological procedures for detection of *Angiostrongylus vasorum*

#### Nina Gillis-Germitsch, Manuela Schnyder

##### Institute of Parasitology, Vetsuisse-Faculty, University of Zurich, Zürich, 8057, Switzerland

###### **Correspondence:** Nina Gillis-Germitsch (nina.gillis@uzh.ch)

*Angiostrongylus vasorum* is a lungworm infecting dogs, foxes and few other wild carnivores [1-3]. Reports of *A. vasorum* in dogs increased in the last two decades and foxes were frequently indicated as the relevant parasite reservoir, together with snails acting as intermediate hosts [4-8]. Our aim was to investigate the prevalence, worm burden and regional distribution of lungworms in Swiss red foxes, as well as to evaluate enzyme-linked immunosorbent assays (ELISA) for detection of circulating *A. vasorum* antigen and specific antibodies, which had previously been developed for dogs [9, 10]. Over the past five years lungs and hearts of 377 Swiss foxes were examined for the presence of *A. vasorum* and other lungworms. Blood collected from these foxes was used to evaluate the ELISAs. In the investigated fox population, *A. vasorum*, *Capillaria aerophila* and *Crenosoma vulpis* were identified: *C. aerophila* was found in all investigated cantons, whereas *A. vasorum* and *C. vulpis* did not occur in the canton of Graubünden. Overall prevalence of *A. vasorum* over the last five years was 45.1% (worm burden, WB: 1-44, mean 7.1), increasing from 20.5% in 2012 to 72.3% in 2016, while overall prevalence of *C. aerophila* and *C. vulpis* was 63.7% (WB: 1-99, mean 3.2) and 9.0% (WB: 1-48, mean 1.2), respectively. The ELISAs for detection of circulating antigen and specific antibodies had a sensitivity and specificity of 91.2% and 89.4%, and of 42.2% and 92.0%, respectively. Cross-reactions with other parasite species were very limited. We therefore present reliable and quick serological methods to detect *A. vasorum* in foxes and conclude that *A. vasorum* is established in the Swiss fox population with increasing prevalence from year to year.

**References**

1. Segovia JM, Torres J, Miquel J, Llaneza L, Feliu C. Helminths in the wolf, *Canis lupus*, from north-western Spain. Journal of Helminthology. 2001; 75(02): 183–192.

2. Bourque A, Whitney H, Conboy G. *Angiostrongylus vasorum* infection in a coyote (*Canis latrans*) from Newfoundland and Labrador, Canada. Journal of Wildlife Diseases. 2005; 41(4): 816–819.

3. Takács A, Szabó L, Juhász L, Lanszki J, Takács P, Heltai M. Data on the parasitological status of golden jackal (*Canis aureus* L., 1758) in Hungary. Acta Veterinaria Hungarica. 2013; 62(1): 33–41.

4. Bolt G, Monrad J, Henriksen P, Dietz HH, Koch J, Bindseil E, Jensen AL. The fox (*Vulpes vulpes*) as a reservoir for canine angiostrongylosis in Denmark. Field survey and experimental infections. Acta Veterinaria Scandinavica. 1992; 33(4): 357–362.

5. Lurati L, Deplazes P, Hegglin D, Schnyder M. Seroepidemiological survey and spatial analysis of the occurrence of *Angiostrongylus vasorum* in Swiss dogs in relation to biogeographic aspects. Veterinary Parasitology. 2015; 212(3–4): 219–226.

6. Tolnai Z, Széll Z, Sréter T. Environmental determinants of the spatial distribution of *Angiostrongylus vasorum*, *Crenosoma vulpis* and *Eucoleus aerophilus* in Hungary. Veterinary Parasitology. 2015; 207(3): 355–358

7. Patel Z, Gill AC, Fox MT, Hermosilla C, Backeljau T, Breugelmans K, Keevash E, McEwan C, Aghazadeh M, Elson-Riggins JG. Molecular identification of novel intermediate host species of *Angiostrongylus vasorum* in Greater London. Parasitology Research. 2014; 113(12): 4363–4369.

8. Guilhon J, Bressou C. Rôle des Limacidés dans le cycle évolutif *d'Angiostrongylus vasorum* (Baillet, 1866). C R Acad Sc. 1960; 251: 2252–2253.

9. Schucan A, Schnyder M, Tanner I, Barutzki D, Traversa D, Deplazes P. Detection of specific antibodies in dogs infected with *Angiostrongylus vasorum*. Veterinary Parasitology. 2012; 185(2-4): 216–224.

10. Schnyder M, Tanner I, Webster P, Barutzki D, Deplazes P. An ELISA for sensitive and specific detection of circulating antigen of *Angiostrongylus vasorum* in serum samples of naturally and experimentally infected dogs. Veterinary Parasitology. 2011; 179(1-3): 152–158.

### A38 Gastropod shedding of third-stage larvae after infection of metastrongyloid lungworms

#### Gary Conboy^1^, Nicole Guselle^1^, Roland Schaper^2^

##### ^1^Department of Pathology and Microbiology, Atlantic Veterinary College, Charlottetown, Prince Edward Island, C1A 4P3, Canada; ^2^Bayer Animal Health GmbH, Leverkusen, Germany

###### **Correspondence:** Gary Conboy (conboy@upei.ca)

Felids and canids acquire infection of metastrongyloid nematode parasites (*Aelurostrongylus abstrusus, Angiostrongylus vasorum, Crenosoma vulpis, Oslerus rostratus, Troglostrongylus* spp.) by the ingestion of infective third-stage larvae (L3) in the tissues of gastropod intermediate hosts (IMH) and in some species also paratenic hosts. Speculation on potential exposure due to L3 shed into the environment has arisen due to reports of L3 released by laboratory aquatic snails experimentally infected *with A. abstrusus, A. vasorum* and *Troglostrongylus brevior*. Spontaneous shedding has not been reported for these species in the terrestrial gastropod natural IMH (slugs, land snails). First-stage larvae of *A. abstrusus*, *A. vasorum* and *C. vulpis* were each placed on lettuce (400 – 1445 L1/slug) and fed to laboratory raised *Limax maximus* in separate exposure groups of 12-42 slugs. In addition, a mixture of *O. rostratus*-*T. wilsoni* (90%/10%) and *T. wilsoni-O. rostratus* (95%/5%) L1 recovered from a co-infected bobcat were used to infect 30 and 22 *L. maximus* (1600 L1/slug), respectively. Slug feces was examined for L3 (2x/week) using a modified Baermann method. All surviving slugs were digested for individual L3 counts at the termination of the studies (105 – 210 days PI). Shedding of L3 was detected in all groups beginning 20-30 days PI and continued from 55 to 202 days PI. No L3 were detected from unexposed control group slugs. Weekly shedding levels for the various infection groups ranged from 0 – 3.273 L3/slug. The percentage of L3 shed ranged from 1.3% (*A. vasorum*) to 2.77% (*C. vulpis*) of the total recovered (= shed L3 + digest L3) from each exposure. Mortality rates in infected slugs ranged from 0% (*A. abstrusus*) to 65% (*Crenosoma vulpis*). Based on identification using morphology, L3 of both *T. wilsoni* and *O. rostratus* were shed in both of the mixture-exposed groups. Longevity in the environment was tested by placing L3 of *A. vasorum* and *T. wilsoni-O. rostratus* on lettuce. Actively motile L3 were recovered from the lettuce for up to 16 days post-deposit. Detection of spontaneous shedding in all 5 parasite species indicates that spontaneous shedding of L3 into the environment is likely a general characteristic of the metastrongyloids. The spontaneous shedding and prolonged survival of L3 indicates that exposure through environmental contamination likely plays a role in infection transmission with these parasite species.

### A39 *Angiostrongylus chabaudi*: first description of the diagnostic stage and confirmation of European wildcat as definitive host

#### Anastasia Diakou^1^, Despina Migli^2^, Angela Di Cesare^3^, Dimitra Psalla^4^, Dionisios Youlatos^2^, Donato Traversa^3^

##### ^1^Laboratory of Parasitology and Parasitic Diseases, School of Veterinary Medicine, Faculty of Health Sciences, Aristotle University of Thessaloniki, Thessaloniki, 54124, Greece; ^2^Department of Zoology, School of Biology, Aristotle University of Thessaloniki, Thessaloniki, 54124, Greece; ^3^Faculty of Veterinary Medicine, Teaching Veterinary Hospital, Località Piano d'Accio snc, Teramo, 64100, Italy; ^4^Laboratory of Pathology, School of Veterinary Medicine, Faculty of Health Sciences, Aristotle University of Thessaloniki, Thessaloniki, 54124, Greece

###### **Correspondence:** Anastasia Diakou (diakou@vet.auth.gr)

**Fig. 6 (abstract A39).**
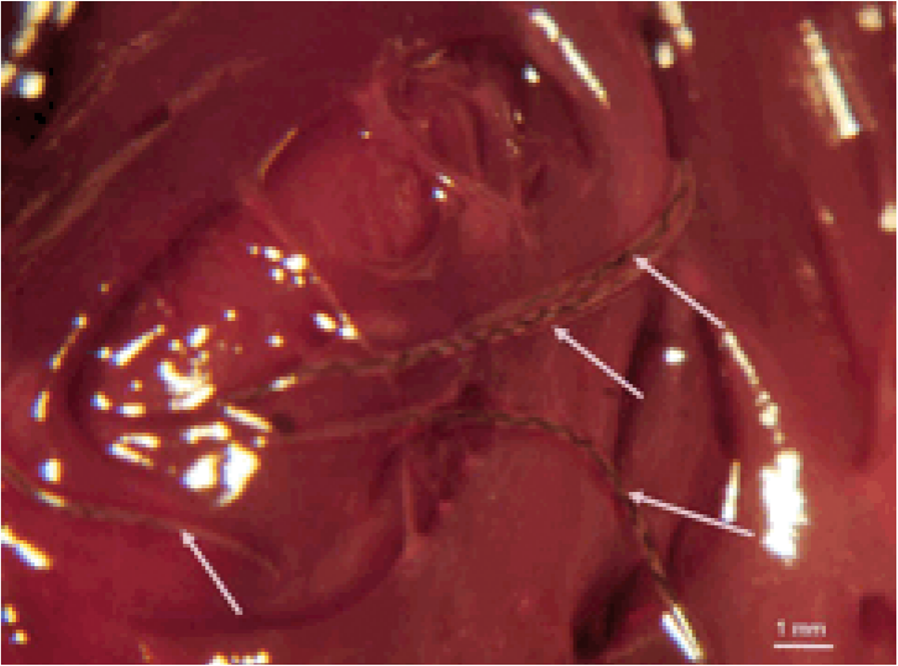
*Angiostrongylus chabaudi* (arrows) on the endothelium of the right ventricle of a wildcat in Northern Greece

**Fig. 7 (abstract A39).**
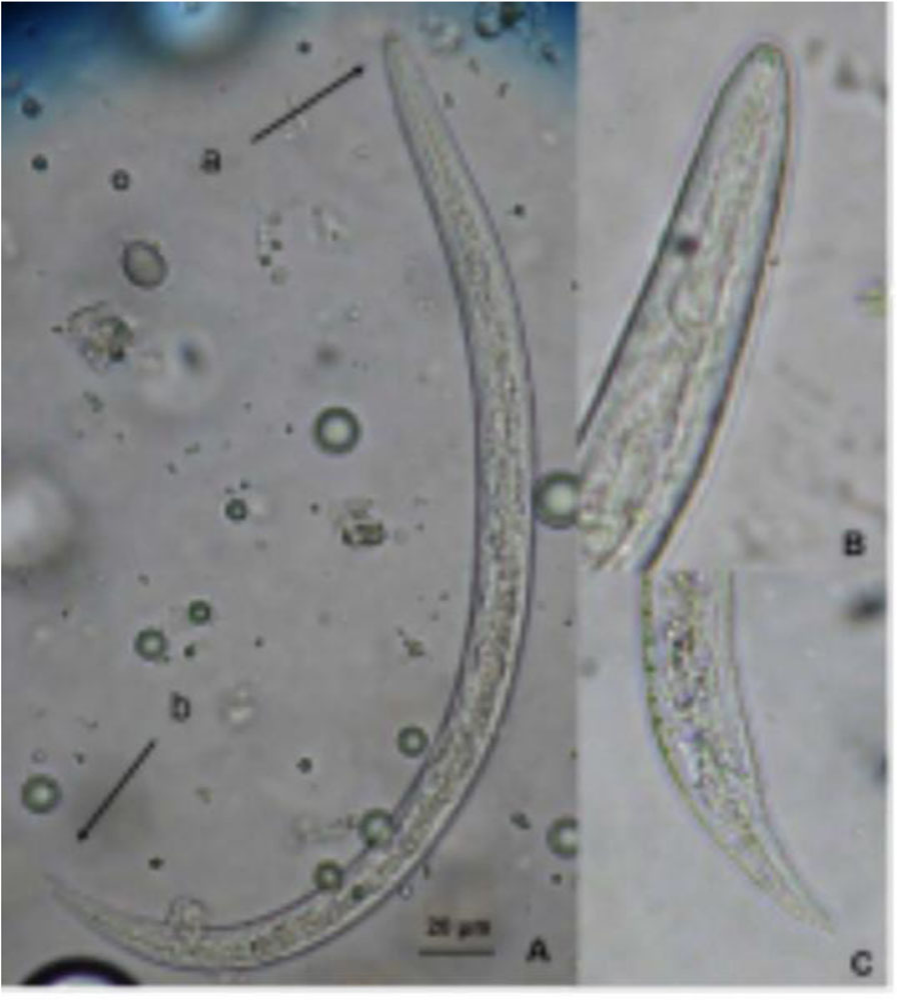
First stage larva (L1) of *Angiostrongylus chabaudi*. A. Whole larva showing anterior (**a**) and posterior extremity (**b**). B. Anterior extremity. C: Posterior extremity

The adult stages of the nematode *Angiostrongylus chabaudi* (Strongylida, Angiostrongylidae), parasitize the pulmonary arteries and right ventricle of the heart and have been reported in wildcats (*Felis silvestris silvestris*) in Italy, in 1957 [1]. Since that first description, *A. chabaudi* has never been reported, with the exception of the recent descriptions of immature stages in two cats in Italy [2, 3]. The case presented here is an infection by *A. chabaudi* in a wildcat from Northern Greece. The wildcat was found road-killed near the lake Kerkini (Macedonia, Greece). During necropsy, nematode parasites were found in the right ventricle of the heart and the pulmonary artery. The parasites were adult males and females and according their morphological characteristics were identified as *A. chabaudi* (Fig. 6)*.* Additionally, parasitological examination of faeces and bronchoalveolar lavage revealed the presence of first stage larvae (L1) measuring 362-400 x 15-18.5 μm, with a kinked tail presenting a dorsal spine and a notch (Fig. 7). Both adults and larvae were subjected to molecular examination that confirmed that the parasites belong to the species *A. chabaudi*. The finding of histopathological examination of the lungs included heavy, extended, interstitial granulomatous pneumonia, with lesions detected around the larvae and eggs of the parasite. These findings were most likely, exclusively due to the presence of *A. chabaudi*, as there were no other parasites found in the lungs, suggesting that this parasite can be quite pathogenic to its hosts. The first description ever [4] of *A. chabaudi* L1 provides the necessary evidence that this nematode can complete its life cycle in the European wildcat, which should be considered its definitive host. The complete life cycle of the parasite remains unknown. For this reason, investigations that will include identification of intermediate hosts (most likely terrestrial molluscs), and development of the parasite both in the vertebrate and invertebrate host, are needed. The description of the diagnostic stage (L1) of *A. chabaudi* provides the basic information for future studies that will investigate infection in other feline species, e.g. the domestic cat and the implications to their health status. It is important to monitor in what extend can *A. chabaudi* affect domestic cats, a scenario that is possible but seems sporadic, according the recent available information of immature, unfertilized, not fully developed parasites isolated from domestic cats.

**References**

1. Biocca E. *Angiostrongylus chabaudi* n. sp. parassita del cuore e dei vasi polmonari del gatto selvatico (*Felis silvestris*). R. Accad. Naz. Lincei. 1957, 22: 526–532.

2. Varcasia A, Tamponi C, Brianti E, Cabras PA, Boi R, Pipia AP, Giannelli A, Otranto D, Scala A. *Angiostrongylus chabaudi* Biocca, 1957: a new parasite for domestic cats? Parasit Vectors., 2014, 7: 588.

3. Traversa D, Lepri E, Veronesi F, Paoletti B, Simonato G, Diaferia M, Di Cesare A. Metastrongyloid infection by *Aelurostrongylus abstrusus*, *Troglostrongylus brevior* and *Angiostrongylus chabaudi* in a domestic cat. Int. J. Parasitol., 2015, 45: 685–690.

4. Diakou A, Psalla D, Migli D, Di Cesare A, Youlatos D, Marcer F, Traversa D. First evidence of the European wildcat (*Felis silvestris silvestris*) as definitive host of *Angiostrongylus chabaudi*. Parasitol. Res., 2016, 115: 1235.

### A40 *Angiostrongylus chabaudi* and *A. daskalovi* in wild carnivores from Romania

#### Călin M Gherman^1^, Georgiana Deak^1^, Angela M Ionică^1^, Gianluca D’Amico^1^, Domenico Otranto^2^, Andrei D. Mihalca^1^

##### ^1^Department of Parasitology, University of Agricultural Sciences and Veterinary Medicine Cluj-Napoca, Calea Mănăștur 3-5, 400372, Cluj-Napoca, Romania; ^2^Dipartimento di Medicina Veterinaria, Università degli Studi di Bari, Bari, Italy

###### **Correspondence:** Andrei D. Mihalca (amihalca@usamvcluj.ro)

*Angiostrongylus chabaudi* is a rare feline cardio-pulmonary nematode, described in 1957 in a wildcat from Italy and reported subsequently in domestic cats from Italy and wildcats from Greece. Similarly, *A. daskalovi* is a cardio-pulmonary nematode of mustelids described in Bulgaria in 1988 and later reported also in badgers from Spain. The present study reports *A. chabaudi* and *A. daskalovi*, in wildcats (*Felis silvestris*) and badgers (*Meles meles*), respectively, collected as roadkills in Romania. After careful morphological and morphometrical identification, the partial mitochondrial cytochrome c oxidase subunit 1 (cox 1) gene and the internal transcribed spacer 2 (ITS2) of the rRNA gene were sequenced and compared with sequences deposited in GenBank. This study reports for the first time in Eastern Europe the presence of *A. chabaudi* and for the first time in Romania the presence of *A. daskalovi*, bringing new insights in their SEM ultrastructure and molecular identification.

## POSTER SESSION

### A41 First characterization of haemocyte extracellular traps in gastropods induced by *Angiostrongylus vasorum, Crenosoma vulpis, Aelurostrongylus abstrusus* and *Troglostrongylus brevior*

#### Malin Lange^1^, Felipe Penagos^1^, Tamara Muñoz-Caro^1^, Gerd Magdowski^2^, Uwe Gärtner^2^, Helena Mejer^3^, Roland Schaper^4^, Carlos Hermosilla^1^, Anja Taubert^1^

##### ^1^Institute of Parasitology, Justus Liebig University, Gießen, Germany, 35392; ^2^Institute of Anatomy and Cell biology, Justus Liebig Universität, Giessen, Germany; ^3^Parasitology and Aquatic Diseases, University of Copenhagen, 1871, Copenhagen, Denmark; ^4^Bayer Health GmbH, Leverkusen, Germany, 51368

###### **Correspondence:** Malin Lange (malin.k.lange@vetmed.uni-giessen.de)

In the last years lungworm infections of canids and felids have been the focus of special attention due to their emergence in several countries and spread into non-reported areas [1, 2, 3]. Slugs and snails have been reported as intermediate hosts of these metastrongylids [4, 5]. Haemocytes, a denomination of cell types in invertebrates that freely circulate in the haemolymph [6], are involved in several physiological functions like coagulation and innate immune response [7]. Haemocytes are similar to mammalian phagocytes, able to produce Extracellular Traps [8]. This phenomenon, denominated ETosis, consists in an programmed cell death form in which the chromatin and antimicrobial proteins are expulsed into the extracellular region and finally induce the formation of fiber-like structures, that have the capacity to trap and inactivate pathogens of diverse kinds, like bacteria, viruses and others parasites [9]. Recently the ETosis mechanism was characterized in invertebrates [8, 10]. The aim of this study was to characterize for the first time gastropod’s Haemocytes Extracellular Traps (HETs) induction and formation against metastrongylid larvae. Haemocytes from the slugs species *Arion lusitanicus* and *Limax maximus*, and the Giant African Snail *Achatina fulica*, were cultured with *Angiostrongylus vasorum*, *Crenosoma vulpis, Aelustrongylus abstrusus* and *Troglostrongylus brevior* L1 larvae as well as L3 larvae of *A. vasorum* at room temperature (±20 °C) and the incubation time varied between 30 min. to 24 h. The visualisation of the HETs was performed using phase contrast microscopy, scanning electron microscopy and fluorescence microscopy. Confronting gastropod haemocytes with the above mentioned species of lungworm larvae revealed in the phase contrast microscopy that L1 and L3 got entangled with a non-defined material originating from the haemocytes. These delicate non-defined ET-like structures were examined in more detail with the technique of SEM imaging. This method renewed our strong suspicion that these structures represent gastropd-derived ETs. Immunofluorescence microscopy revealed that these structures contain histones and DNA. Which have been proven to play an important role in the process of ETosis [11, 12]. This survey represents first indications on slugs and snails casting HETs. All methods used to visualize Extracellular Trap-formation provided strong evidence that this innate immune defence mechanism also exists in gastropods.

**References**

1. Helm, J., Roberts, L., Jefferies, R., Shaw, S. E., Morgan, E. R. Epidemiological survey of Angiostrongylus vasorum in dogs and slugs around a new endemic focus in Scotland. The Veterinary record. 2015; 177

2. Taubert, A., Pantchev, N., Vrhovec, M.G., Bauer, C., Hermosilla, C. Lungworm infections (*Angiostrongylus vasorum, Crenosoma vulpis, Aelurostrongylus abstrusus*) in dogs and cats in Germany and Denmark in 2003-2007. Veterinary parasitology. 2009; 159:175–180.

3. Traversa, D., Di Cesare, A., Conboy, G. Canine and feline cardiopulmonary parasitic nematodes in Europe: emerging and underestimated. Parasites & vectors. 2010; 623: 62.

4. Ferdushy, T., Hasan, M.T. *Angiostrongylus vasorum*: the 'French Heartworm'. Parasitology research. 2010 107:765-771.

5. Patel, Z., Gill, A. C., Fox, M. T., Hermosilla, C., Backeljau, T., Breugelmans, K., Elson-Riggins, J. G. Molecular identification of novel intermediate host species of *Angiostrongylus vasorum* in Greater London. Parasitology research. 2014; 113:4363–4369.

6. Yoshino, T. P., Wu, X. J., Gonzalez, L. A., & Hokke, C. H. Circulating *Biomphalaria glabrata* hemocyte subpopulations possess shared schistosome glycans and receptors capable of binding larval glycoconjugates. Experimental parasitology. 2013; 133: 28–36.

7. Cheng, T. C., & Sullivan, J. T. Effects of heavy metals on phagocytosis by molluscan hemocytes. Marine Environmental Research. 1984; 14:305–315.

8. Robb, C. T., Dyrynda, E. A., Gray, R. D., Rossi, A. G., & Smith, V. J. Invertebrate extracellular phagocyte traps show that chromatin is an ancient defence weapon. Nature communications. 2014; 5.

9. Brinkmann, V., Goosmann, C., Kühn, L. I., & Zychlinsky, A. Automatic quantification of in vitro NET formation. Frontiers in immunology. 2012; 3.

10. Poirier, A. C., Schmitt, P., Rosa, R. D., Vanhove, A. S., Kieffer-Jaquinod, S., Rubio, T. P., Destoumieux-Garzón, D. Antimicrobial Histones and DNA Traps in Invertebrate Immunity: Evidences in *Crassostrea Gigas*. Journal of Biological Chemistry. 2014; 289:24821–24831.

11. Brinkmann, V., U. Reichard, C. Goosmann, B. Fauler, Y. Uhlemann, D.S. Weiss, Y. Weinrauch, A. Zychlinsky. Neutrophil extracellular traps kill bacteria. Science. 2004; 303:1532–1535.

12: Urban, C.F., D. Ermert, M. Schmid, U. Abu-Abed, C. Goosmann, W. Nacken, V. Brinkmann, P.R. Jungblut, A. Zychlinsky. Neutrophil extracellular traps contain calprotectin, a cytosolic protein complex involved in host defense against *Candida albicans*. PLoS Pathog. 2009; 5

### A42 Assessment of recovery rates and morphology of larvae *A. abstrusus* in flotation methods using five solutions with different specific gravities (S.G.)

#### Klaudiusz Szczepaniak^1^, Krzysztof Tomczuk^1^, Maciej Grzybek^1^, Ryszard Iwanicki^2^

##### ^1^Department of Parasitology and Invasive Diseases, University of Life Sciences, Lublin, ul. Akademicka 12, 20-950 Lublin, Poland; ^2^Lubelskie Centrum Małych Zwierząt, ul. Stefczyka 11, 20-151 Lublin, Poland

###### **Correspondence:** Klaudiusz Szczepaniak (k.o.szczepaniak@up.lublin.pl)

The most prevalent cardiopulmonary nematodes in domestic cats in Europe is *Aelurostrongylus abstrusus* [1]. Recently, Baermann migration method is the gold standard for the diagnosis of lungworms invasions, but takes 24 h and requires at least 4 g of fresh fecal samples. Furthermore, taxonomical classification of motile larvae may be difficult, because their identification is particular based on the tail shape [2]. Unlike the Baermann method flotation-based techniques are easy to performed, fast and allow to detected wide range of parasites. Fecal samples could be delivered to the laboratory preserved or frozen. Specific gravities of different flotation fluid as well as exposures time resulted in the number and morphologic deformations of the larvae derived from each methods modification [3]. The aim of this study was to assessment of the recovery rates and morphology of larvae *A. abstratus* in flotation methods using five fluids with different specific gravities (S.G.) Fresh fecal sample (6 g) from natural infected with *A. abstrusus* cat (three years old, male, previously not treated) were obtained. The number of lungworm larvae per gram of feces (LPG) was estimated - 2800/g, using modified Baermann methods and McMaster chambers. Subsequently, five flotation with different flotation solutions respectively: 33% ZnSO_4_ (SG 1.18)_,_ saturated NaCl (SG 1.20), commercially available NaNO_3_ - Fecasol (SG 1.20), saturated NaCl and sacharose (SG 1.25), saturated NaNo_3_ (SG 1.33) were performed. We used the following procedure: 1 g fecal sample was mixed with 35 ml flotation solution and poured through a strainer into a (25 ml) glass Erlenmeyer Flask. Slides were directly analyzed under light microscope with Nomarski contrast. A total number of larvae (recovery rates) for each flotation was estimated. Simultaneously larvae were recorded as identifiable (tail was visible) or unidentifiable (tail was not visible e.g., morphologic deformations or curled larvae). Statistical data analysis was performed using Analysis ToolPak Microsoft Office Excel. The larvae of *A. abstrusus* were found in all flotations. The biggest recovery rates 3.2 and 3.1 were achieved using flotation solutions with the highest specific gravities (saturated solutions of: NaCl/sacharose and NaNo3). In solutions with SG from 1.18 to 1.2 number of detected larvae were lower but their characterized by high percentage of identifiable larvae ranged from 56.3% (saturated NaCl), 71.4% (Fecasol) to 85.7% (33% ZnSO4). Details of results are presented in Table 2.

**References**

1. Barutzki D, Schaper R. Occurrence and regional distribution of *Aelurostrongylus abstrusus* in cats in Germany. Parasitol Res.2013; 112:855–861.

2. Ribeiro VM, Lima WS. Larval production of cats infected and re-infected with *Aelurostrongylus abstrusus* (*Nematoda: Protostrongylidae*). Rev Med Vet. 2001; 152: 815–820.

3. Lima VF, Cringoli G, Rinaldi L, Monteiro MF, Calado AM, Ramos RA, Meira-Santos PO, Alves LC. A comparison of mini-FLOTAC and FLOTAC with classic methods to diagnosing intestinal parasites of dogs from Brazil. Parasitol Res. 2015; 114: 3529–3533.

**Table 2:** A Comparison of various flotation fluids in *A. abstrusus* larvae (L1) detection

flotation solutions SG - specific gravities	recovery rates for LPG 2800	number of larvae in flotation	identifiable	unidentifiable	% identifiable	average lenght of larvae
33% ZnSO4 (SG 1.18)	0.5	14	12	2	85.7	340.6
saturated NaCl (SG 1.20)	1.1	32	18	14	56.3	354.9
commercially available NaNO3 - Fecasol (SG 1.20)	1.0	28	20	8	71.4	359.2
saturated NaCl and sacharose (SG 1.25)	3.2	90	28	62	31.1	374.2
saturated NaNo3 (SG 1.33)	3.1	86	20	66	23.3	383.8

### A43 A ten-year retrospective study of angiostrongylosis at Alfort Veterinary School, Ile de France

#### Benjamin Bedel^1^, Radu Blaga^2^, Vassiliki Gouni^3^, Valérie Chetboul^3^, Ghita Benchekroun^4^, Stéphane Blot^4^, Patrick Verwaerde^1^, Bruno Polack^2^

##### ^1^Department of Emergency and intensive care, Centre Hospitalier Universitaire. Université Paris-Est, Ecole Nationale Vétérinaire d’Alfort, 94704 Maisons-Alfort, France; ^2^Department of parasitology, BioPôle. Université Paris-Est, Ecole Nationale Vétérinaire d’Alfort, 94704 Maisons-Alfort, France; ^3^Department of Cardiology, Centre Hospitalier Universitaire. Université Paris-Est, Ecole Nationale Vétérinaire d’Alfort, 94704 Maisons-Alfort, France; ^4^Department of Small Animal Internal Medicine, Centre Hospitalier Universitaire. Université Paris-Est, Ecole Nationale Vétérinaire d’Alfort, 94704 Maisons-Alfort, France

###### **Correspondence:** Bruno Polack (bruno.polack@vet-alfort.fr)

A retrospective study was conducted for the period of 2005-2014, on the identified cases of angiostrongylosis within the clinics of Alfort Veterinary School which receive around 9000 dogs per year. During the ten-year period, the research for angiostrongylosis was done on 804 animals aging from 2 month to 18 year old (mean age = 5.6 years). Three different parasitological methods were performed: faecal examination by Baermann technic (mainly on faeces of three consecutive days), broncho-alveolar lavage (BAL) direct observation by binocular microscopy and *Angiostrongylus* antigen detection test (IDEXX Angio Detect™ Test; used only in 2014), respectively on 718, 150 and 3 dogs. Some dogs were tested by two different methods. Infection by *Angiostrongylus vasorum* was detected in thirty dogs, corresponding to 3.7% of tested animals. Infected dogs were aged from 4 months to 16-year-old (mean age = 5.9 years). Positive results were observed on 30 Baermann tests (4.2% of positive) and 1 BAL examination (0.7%, this animal was also tested by Baermann). Concerning the annual dynamic of identified cases, except for 2009, 2010 and 2014, when respectively 5, 4 and 1 case have been identified, for the rest of the period, an annual 3 cases identification rate was observed. The detection was higher during the first 5 months of the year, 5.4% versus 2.3% for the 7 last months. The clinical observed symptoms were very variable: dyspnoea, coagulopathy, right-sided heart failure, cutaneous *larva migrans*.

### A44 Prevalence of *Aelurostrongylus abstrusus* in Danish cats

#### Alice P. Hansen^1^, Lene M. Vinther^1^, Line K. Skarbye^1^, Caroline S. Olsen^2^, Helena Mejer^1^, Jakob L. Willesen^3^

##### ^1^Department of Veterinary Disease Biology, University of Copenhagen, 1870 Frb C. Denmark; ^2^Environmental Support, DFP, Novo Nordisk, 4400 Kalundborg, Denmark; ^3^Department of Veterinary Clinical and Animal Sciences, University of Copenhagen, 1870 Frb C., Denmark

###### **Correspondence:** Jakob L. Willesen (jw@sund.ku.dk)

*Aelurostrongylus abstrusus* is considered the most prevalent lungworm worldwide in domesticated cats [1, 2]. High prevalence rates have especially been found in southern Europe [3, 4] and studies have indicated that the infection is of clinical relevance [5, 6, 7]. A recent study revealed a high occurrence of *A. abstrusus* in euthanized cats from eastern Denmark [8] which raised concern of an underestimated national prevalence. Based on these findings, the objective of the present study was to investigate the national prevalence of *A. abstrusus* in Danish cats. For this purpose, faecal samples from 327 cats were collected between August and October 2015. The study population consisted primarily of outdoor cats from shelters distributed across Denmark and a modified Baermann method was used to test for the infection. The national prevalence of *A. abstrusus* was 8.3% [95% CI: 5.6-11.9] with substantial regional variation. In Northern Jutland the prevalence was 0% [95% CI: 0.0-8.8] while a prevalence of 31.4% [95% CI: 16.9-49.3] was found in Western Jutland. The prevalence in the remaining regions varied from 4.5-9.7%. Living in rural areas was identified as a risk factor for infection with *A. abstrusus* (p = 0.0001) and this accounted for most of the variation in regional prevalence. Aelurostrongylosis was not detected in cats younger than 11 weeks and the prevalence in this age group was significantly lower than in older cats (p = 0.002). Based on these findings, lactogenic transmission seems unlikely, despite the fact that this route has been suggested for the closely related feline lungworm *Troglostrongylus brevior* [9]. The results of the present study demonstrated that *A. abstrusus* is endemic in Denmark. Therefore, this parasite should be considered an important differential diagnosis in any Danish cat displaying respiratory symptoms. The infection is especially relevant in outdoor cats living in rural areas. Other than rural origin, differences in regional prevalence may result from factors influencing the presence of intermediate and transport hosts, such as climate. However, socioeconomic differences between regions may also in part explain the differences in the current prevalence rates. With increased movement of pets, more extensive testing for *A. abstrusus* is warranted to monitor the distribution and prevalence of *A. abstrusus*.

**References**

1. Barutzki D. and Schaper R. 2013. Occurrence and regional distribution of *Aelurostrongylus abstrusus* in cats in Germany. *Parasitol. Res.* 112(2):855–861.

2. Traversa, D. and Cesare A. 2013. Feline lungworms: what a dilemma. *Trends Parasitol.* 29(9):423–430.

3. Payo-Puente, P., Botelho-Dinis M, Uruena AMC, Payo-Puente M, Gonzalo-Orden JM and Rojo-Vazquez FA. 2008. Prevalence study of the lungworm *Aelurostrongylus abstrusus* in stray cats of Portugal. *J Fe Med Surg.* 10(3):242–246.

4. Traversa, D. and Guglielmini C. 2008. Feline aelurostrongylosis and canine angiostrongylosis: a challenging diagnosis for two emerging verminous pneumonia infections. *Vet. Parasitol.* 157(3/4):163–174.

5. Di Cesare, A., Di Francesco G, Di Regalbono AF, Eleni C, De Liberato C, Marruchella G, Iorio R, Malatesta D, Romanucci MR, Bongiovanni L, Cassini R and Traversa D. 2015. Retrospective study on the occurrence of the feline lungworms *Aelurostrongylus abstrusus* and *Troglostrongylus spp.* in endemic areas of Italy. *Vet. J.* 203(2):233–238.

6. Genchi, M, Ferrari N, Fonti P, De Francesco I, Piazza C and Viglietti A. 2014. Relation between *Aelurostrongylus abstrusus* larvae excretion, respiratory and radiographic signs in naturally infected cats. *Vet. Parasitol.* 206(3/4):182–187

7. Traversa, D., Lia RP, Lorio R, Boari A, Paradies P, Capelli G, Avolio S and Otranto D. 2008. Diagnosis and risk factors of *Aelurostrongylus abstrusus* (Nematoda, Strongylida) infection in cats from Italy. *Vet. Parasitol.* 153(1/2):182–186.

8. Olsen CS, Willesen JL, Pipper CB and Mejer H. 2015. Occurrence of *Aelurostrongylus abstrusus* (Railliet, 1898) in Danish cats: a modified lung digestion method for isolating adult worms. *Vet. Parasitol.* 210(1/2):32–39.

9. Brianti E, Gaglio G, Napoli E, Falsone L, Giannetto S, Latrofa MS, Giannelli A, Dantas-Torres F and Otranto D. 2013. Evidence for direct transmission of the cat lungworm *Troglostrongylus brevior* (Strongylida: Crenosomatidae). *Parasitology.* 140(7):821–824.

### A45 Unusual clinical cases of *Angiostrongylus vasorum* infections

#### Angela Di Cesare^1^, Luigi Venco^2^, Simone Manzocchi^3^, Eleonora Grillotti^1,4^, Edoardo Auriemma^5^, Fabrizio Pampurini^6^, Cecilia Garofani^4^, Fabrizio Ibba^7^, Donato Traversa^1^

##### ^1^Faculty of Veterinary Medicine, University of Teramo, 64100, Teramo, Italy; ^2^Veterinary Hospital "Città di Pavia", Viale Cremona 179, 27100, Pavia, Italy; ^3^Novara Day Lab - IDEXX Laboratories Italia Granozzo con Monticello, Italy; ^4^Veterinary Pratice "Centro Italia", Via Biancifiori 3, 02100, Rieti, Italy; ^5^Istituto Veterinario di Novara, S.P. 9, 28060, Granozzo con Monticello, Italy; ^6^Bayer Sanità Animale, Viale Certosa 130, 20156, Milan, Italy; ^7^Veterinary Pratice "Poggio dei Pini" Strada 40, 09012, Capoterra, Cagliari, Italy

###### **Correspondence:** Angela Di Cesare (angdicesare@gmail.com)

In dogs *Angiostrongylus vasorum* often causes a severe infection characterized by varying signs similar to those of other canine diseases. Although this parasitosis may be life-threatening, dog angiostrongylosis is often underestimayed and veterinarians do not use appropriate diagnostic tests. Six cases of angiostrongylosis are here described, with a focus on their clinical features, that were unusual and confounding. Although the six were refereed with clinical signs that may occur in the infection by *A. vasorum*, the animals were suspected to have other diseases before a correct diagnosis was achieved. Case 1: a dog showed clinical, radiographic and ultrasound features compatible with a pulmonary tumour; case 2: this dog was simultaneously infected by *A. vasorum* and *Dirofilaria immitis* but the former nematode was not included in the differential diagnosis ; case 3: a critically ill dog was referred for a severe and then fatal dyspnoea of initially unknown origin; case 4: a thrombocytopenia recorded in a dog with hemorrhages and ecchymoses was erroneously attributed to an inherited, immune-mediated or infective origin; Case 5: a discospondylitis was considered to be the cause of neurological signs in a dog; case 6: a cardio-pulmonary dirofilariosis was diagnosed in a dog that had, on the contrary, only angiostrongylosis. A prompt administration of a parasiticide (in most cases topical moxidectin) was efficacious in the treatment of *A. vasorum* infection in dogs n. 1, 2, 4 and 5, i.e. those animals that did not show hazardous lung haemorrhages yet at the referral. Currently, canine angiostrongylosis is spreading in various regions for different biological and epizootiological factors. Importantly, animals n. 1-5 were diagnosed with *A. vasorum* in italian regions where this parasite is not considered endemic. Thus, it is of importance that practitioners must include *A. vasorum* in the differential diagnosis of any clinical picture compatible with dog angiostrongylosis also when the parasite is not expected to occur and/or in the presence of compatible signs even if the clinical picture is atypical. These clinical cases are described in detail in ref. [1].

**References**

[1] Di Cesare A, Traversa D, Manzocchi S, Meloni S, Grillotti E, Auriemma E, Pampurini F, Garofani C, Ibba F, Venco L. Elusive *Angiostrongylus vasorum* infections. Parasit Vectors. 2015; 8:438.

### A46 First report of *Angiostrongylus vasorum* infections in dogs as well as in the neozoan intermediate host (*Achatina fulica*) in Medellín, Colombia

#### Felipe Penagos^1,2^, Jesed Gutiérrez^1^, Juan D. Velez^1^, Diego Piedrahita^1^, Malin Lange^2^, Carlos Hermosilla^2^, Anja Taubert^2^, Jenny Chaparro^1^

##### ^1^CIBAV research group, Veterinary Medicine School, Faculty of Agrarian Sciences, University of Antioquia, Medellín, Antioquia, 050034, Colombia; ^2^Institute of Parasitology, Justus Liebig University Giessen, Giessen, 35392, Germany

###### **Correspondence:** Felipe Penagos (Felipe.penagos@udea.edu.co)

*Angiostrongylus vasorum* is considered as one of the most pathogenic species of the cardiopulmonary system of wild and domestic canids worldwide. As such, in 1961 this metastrongyloid parasite was detected in South America in crab-eating foxes (*Cerdocyon thous*) in Colombia and in domestic dogs in Brazil. These reports in demonstrated clearly the presence of this parasite in South America. Nonetheless, since then very little has been published on *A. vasorum* infections neither in wild canids nor domestic dogs in South America and Colombia. Thus, aim of this study was to gain current knowledge on the presence of *A. vasorum* in domestic dogs as well as neozoan intermediate hosts by analysing dog faecal samples collected in public parks in Medellin city and from collected neozoan terrestrial snails (*Achatina fulica*). In total 364 faecal samples were collected from February to April 2016, analysed thereafter by Bearmann funnel test with submersion of the samples for at least 24 h. Additionally 300 *A. fulica* snails were collected and digested for the presence of *A. vasorum* larvae The larvae obtained from molluscs were identified on the basis of morphological findings of the tail according to Georgi and Georgi (1991). In total 0.27% of faecal samples contained vital *A. vasorum* larvae (L1) and 2.66% of snails were infected with *A. vasorum*-larvae. Overall, these results show for the first time canine *A. vasorum* infections and also the presence of infected snails thereby proofing the capability of this metastrongyloid nematode to adapt to new emerging intermediate hosts in Colombia. Thus, more research on epidemiology and biology of this neglected parasite and other closely related metastrongyloid nematodes with zoonotic potential are urgently needed in Colombia.

### A47 Canine filarial and *A. vasorum* infections in an area of Central Italy (border Tuscany-Latium)

#### Fabio Macchioni^1^, Marta Magi^1^, Elisa Ulivieri^1^, Francesca Gori^1^, Manuela Schnyder^2^

##### ^1^Department of Veterinary Science, University of Pisa, Viale delle Piagge 2, 56124 Pisa, Italy; ^2^Institute of Parasitology, Vetsuisse Faculty, University of Zurich, 8057 Zurich, Switzerland

###### **Correspondence:** Fabio Macchioni (fabio.macchioni@unipi.it)

Canine filarial infections are widespread throughout the world. New cases in dogs and in humans are occurring in many countries that were previously considered to be free or for which epidemiological data were not available [1]. *Angiostrongylus vasorum* is a cardiopulmonary parasite of wild and domestic canids. In Europe it is widespread in foxes, whereas in dogs it is “emergent” [2]. Both, filariae and *A. vasorum,* are increasingly reported in dogs in overlapping areas [3]. The aim of this work was to determine the occurrence of different species of filarial nematodes in dogs in an area of Central Italy at the border of two regions Tuscany-Latium, traditionally considered free and where epidemiological data in literature are lacking. Also the occurrence of *A. vasorum* was never investigated in this area. In the years 2015-2016 blood samples were collected from 100 dogs living in rural areas at the border between Tuscany and Latium, 50 sera from the province of Grosseto (Tuscany) and 50 from the province of Viterbo (Latium), respectively, and submitted to Knott’s test and ELISA for *Dirofilaria immitis* antigen detection (Dirocheck, Synbiotics®). Furthermore, 56 of these 100 dog samples were serologically tested for *A. vasorum* by ELISAs [4, 5]. Overall 54/100 dogs were positive for microfilariae. In Tuscany 17 out of 50 dogs (34%) were positive for dirofilariosis, i.e. 11 (22%) dogs were positive for *Dirofilaria immitis* and 6 (12%) for *Dirofilaria repens*. In Latium 10 out of 50 dogs (20%) were positive for dirofilariosis, of which 7 (14%) dogs were positive for *D. immitis,* 2 (4%) for *D. repens* and one dog (2%) had a concurrent infection with *D. immitis* and *D. repens*. Morphological identifications were confirmed by histochemical staining. Serological analysis for *A. vasorum* identified 3 cases (0.6%) originating from the Latium region, 2 of which were positive also for *D. immitis*. The results of this study highlight that canine filarial infections are expanding in previously considered free areas in Italy, as it is happening in many other countries. Single seropositive cases of *A. vasorum* anticipate the occurrence of this parasite in this area never investigated before. The presence of filarial infections in dogs suggests the need for prophylaxis in the study area, where it is actually not routinely performed.

**References**

1. Simón F, Siles-Lucas M, Morchón R, González-Miguel J, Mellado I, Carretón E, Montoya-Alonso JA. Human and animal dirofilariosis: the emergence of a zoonotic mosaic. Clin Microbiol Rev 2012; 25: 507–544.

2. Taylor CS, Gato RG, Learmount J, Aziz NA., Montgomery C, Rose H, Wall R. Increased prevalence and geographic spread of the cardiopulmonary nematode *Angiostrongylus vasorum* in fox populations in Great Britain. Parasitology 2015; 142(09): 1190–1195.

3. Del Prete L, Maurelli MP, Pennacchio S, Bosco A, Musella V, Ciuca L, Rinaldi L. *Dirofilaria immitis* and *Angiostrongylus vasorum*: the contemporaneous detection in kennels. BMC Vet Res 2015; 11(1): 1.

4. Schnyder M, Tanner I, Webster P, Barutzki D, Deplazes P. An ELISA for sensitive and specific detection of circulating antigen of *Angiostrongylus vasorum* in serum samples of naturally and experimentally infected dogs. Vet Parasitol 2011; 179: 152–158.

5. Schucan A, Schnyder M, Tanner I, Barutzki D, Traversa D, Deplazes P. Detection of specific antibodies in dogs infected with *Angiostrongylus vasorum*. Vet Parasitol 2012; 185: 216–224.

